# Clinical implementation, barriers, and unmet needs of rTMS and neuro-navigation systems in stroke rehabilitation: a nationwide survey in South Korea

**DOI:** 10.3389/fneur.2024.1423013

**Published:** 2024-07-30

**Authors:** Ga Hui Yu, Chulmin Park, Myeong Geun Jeong, Gun Seo Jung, Kyoung Tae Kim

**Affiliations:** Department of Rehabilitation Medicine, Keimyung University Dongsan Hospital, Keimyung University School of Medicine, Daegu, Republic of Korea

**Keywords:** repetitive transcranial magnetic stimulation, neuro-navigation system, clinical unmet need, nationwide survey, stroke

## Abstract

**Objective:**

The objective of this study was to determine the implementation, clinical barriers, and unmet needs of repetitive transcranial magnetic stimulation (rTMS) and neuro-navigation systems for stroke rehabilitation.

**Design:**

We employed a nationwide survey via Google Forms (web and mobile) consisting of 36 questions across rTMS and neuro-navigation systems, focusing on their implementation, perceptions, and unmet needs in stroke recovery. The survey targeted physiatrists registered in the Korean Society for Neuro-rehabilitation and in rehabilitation hospitals in South Korea.

**Results:**

Of 1,129 surveys distributed, 122 responses were analyzed. Most respondents acknowledged the effectiveness of rTMS in treating post-stroke impairments; however, they highlighted significant unmet needs in standardized treatment protocols, guidelines, education, device usability, and insurance coverage. Unmet needs for neuro-navigation were also identified; only 7.4% of respondents currently used such systems, despite acknowledging their potential to enhance treatment accuracy. Seventy percent of respondents identified lack of prescription coverage, time and errors in preparation, and device cost as barriers to clinical adoption of neuro-navigation systems.

**Conclusion:**

Despite recognition of the potential of rTMS in stroke rehabilitation, there is a considerable gap between research evidence and clinical practice. Addressing these challenges, establishing standardized protocols, and advancing accessible neuro-navigation systems could significantly enhance the clinical application of rTMS, offering a more personalized, effective treatment modality for stroke recovery.

## 1 Introduction

Stroke is the second leading cause of death, despite advancements in acute stroke treatment (1). The most significant impacts of stroke on patients and caregivers are long-term disability, activity limitation, and reduced social participation (2). Approximately two-thirds of patients with stroke are unable to return to their jobs due to persistent motor and cognitive impairment (3).

Neuroplasticity is an adaptive structural and functional change that occurs in the brain and induces cortical reorganization as an important process mediating functional recovery after stroke (4). Recently, neuromodulation has been applied to harness neural plasticity for faster and better recovery. Repetitive transcranial magnetic stimulation (rTMS), a form of neuromodulation, is a non-invasive brain stimulation technique that can modulate human cortical excitability (5).

Transcranial magnetic stimulation (TMS) is based on the generation of a magnetic field by a magnetic coil, which initiates the flow of ions and changes the electrical charge on cell membranes in the brain cortex, resulting in neuronal depolarization or hyperpolarization (6). A specific, repetitive pattern of TMS, rTMS, induces synaptic changes, such as long-term potentiation and depression, and alteration of cortical excitability, facilitating plasticity to improve motor recovery after stroke (7, 8).

Previous studies indicate that rTMS is effective for various post-stroke impairments. For example, contra-lesional low-frequency (1 Hz) rTMS at the primary motor cortex improves motor weakness after a stroke (3). Continuous theta burst stimulation at the parietal cortex significantly improves symptoms in patients with hemispatial neglect (9). Regarding language dysfunction, low-frequency (1 Hz) rTMS over the right inferior frontal gyrus has positive impacts on naming accuracy (10). Nonetheless, there is a lack of consistency in protocols regarding whether rTMS was administered in conjunction with speech-language therapy (SLT) and, if so, the intensity and type of SLT provided (11, 12). This inconsistency has been pointed out as a limitation that lowers the quality of research in this field (13). Furthermore, although a recent meta-analysis indicated that both ipsilesional high-frequency and contralesional low-frequency rTMS may be effective for treating post-stroke swallowing difficulties (14), it is imperative to address and rectify the previously inherent methodological flaws in future studies to establish more robust and compelling evidence (15).

Typically, it has a high level of clinical evidence for the treatment of psychiatric diseases, including depression, anxiety, obsessive-compulsive disorder, and addictions. Furthermore, it has been widely used in clinical practice since obtaining several Food and Drug Administration (FDA) approvals. While numerous randomized controlled studies (7, 8, 16) and interventions have been conducted in post-stroke rehabilitation over several decades, rTMS is often underutilized in clinical practice. The limited clinical utilization of rTMS can be attributed to various factors. One of the primary issues is the absence of standardized clinical guidelines for the use of rTMS in stroke patients. Moreover, concerns regarding the accuracy and reliability of treatment administration further contribute to the hesitancy among healthcare professionals. Regulatory hurdles and reimbursement challenges also pose significant barriers to the widespread adoption of rTMS in clinical settings. In South Korea, only FDA-approved depression is considered for rTMS under non-reimbursement, excluding stroke. Likewise, in Japan and many European countries, rTMS is not covered by medical insurance or reimbursed, restricting access within public health systems (17, 18). In the United States, repeated courses of TMS are not routinely covered by insurance, and there are significant obstacles to obtaining coverage through insurance (19), limiting the accessibility and availability of treatment for underserved patients (20).

Consequently, these factors collectively diminish physicians’ confidence and willingness to actively integrate rTMS into their treatment regimens, ultimately leading to its underutilization in stroke rehabilitation. However, as of yet, no comprehensive investigation of unmet needs among physiatrists applying rTMS for stroke recovery in clinical practice has been conducted.

The primary objective of this study was to investigate the current status, clinical practice implementation, perceptions, and barriers related to rTMS therapy and neuro-navigation in stroke rehabilitation through a nationwide survey. Additionally, we sought to pinpoint the unmet clinical needs for the application of these treatments. We aimed to provide a comprehensive overview of the current landscape and highlight the potential areas for enhancement in the application of rTMS and neuro-navigation. We expect to enhance the level of evidence for rTMS therapy and develop new indications and treatment techniques by identifying research topics that address the unmet needs. Moreover, advancements in rTMS devices and software have the potential to boost the treatment’s convenience, accuracy, and safety.

## 2 Methods

### 2.1 Survey instrument

The survey instrument was developed based on a comprehensive review of existing literature on rTMS treatment in stroke rehabilitation and in-depth interview with leading experts. In developing the questionnaire, key references included recent meta-analyses and clinical guidelines for rTMS treatment (7, 21–23). To ensure quality and reliability, the development process followed the CHERRIES checklist and included several key steps (22). Three physiatrists who specialize in the clinical use and research of rTMS jointly developed the questionnaire. The preliminary version of the questionnaire was administered to two experts in rehabilitation medicine for pre-testing. This process ensured, first, comprehension and interpretation of questions and response items; second, flow, salience, complexity of the questions, and the number of items; third, identification of missing items or response options; and fourth, time required to complete the survey. This feedback was meticulously analyzed and utilized to refine and enhance the survey instrument, thereby ensuring its validity and reliability.

The survey was comprised of 36 optional or open-ended questions, categorized into three distinct domains: (1) demographics of respondents, (2) questions related to rTMS, and (3) questions related to neuro-navigation systems. Current state, implementation, perceptions, barriers, and unmet needs of rTMS therapy for stroke treatment and the neuro-navigation system were investigated with a questionnaire consisting of a Likert-like scale or multiple-choice items. The full description of the questionnaire used in the survey is provided in Supplementary material 1. The survey was administered via Google Forms, which is accessible through both web and mobile platforms. To improve the response rate, emails and postal mailings were used.

### 2.2 Participants

In South Korea, the majority of rTMS treatments for stroke patients are administered by physiatrists in rehabilitation departments. To enhance the consistency and quality of survey responses, we exclusively recruited physiatrists who consented to participate in the survey and provide their personal information for research purposes. Before respondents began the questionnaire, written informed consent was obtained online. To mitigate the risk of duplicate responses, the survey required both the email address and medical license number of the respondent. All identifying information was subsequently anonymized during data analysis to ensure privacy. This study was approved by the Institutional Review Board of Keimyung University Dongsan Medical Center (IRB No. 2023-08-005). This study followed the STROBE guidelines and reports the required information accordingly (see Supplementary Table 1).

### 2.3 Intervention

The initial round of the survey was conducted via email, including a survey link to registered members of the Korean Society for Neuro-rehabilitation. To expand the range of survey responses, a second round was conducted by postal survey, including a QR code linked to the online survey to 52 “designated rehabilitation hospitals.” A designated rehabilitation hospital, officially authorized by the Ministry of Health and Welfare, is representative of rehabilitation hospitals in South Korea and meets the national standards of facilities, human resources, equipment, and medical services. In addition, 22 regional rehabilitation hospitals in the local area were included to identify the characteristics unique to provincial hospitals. The survey was conducted from October 23, 2023, to December 2, 2023.

### 2.4 Statistical analysis

We used descriptive analyses to summarize the participants’ demographics. To further specify the characteristics of the respondents and the results of the survey, age, type of hospital, job title, and years of board certification as a physiatrist were categorized, as shown in Table 1. We also used descriptive statistics, such as frequencies and percentages, to analyze the survey results regarding rTMS and neuro-navigation systems. For open-ended questions, we grouped similar responses to itemize them to facilitate data interpretation. Then the proportions for each item were organized. Statistical analyses were conducted using SPSS version 21.0 (IBM Corp., Armonk, NY, United States).

**Table 1:** Demographics of respondents.

Demographics		Total (*n* = 122)
Sex	Male	88 (72.1)
	Female	34 (27.9)
Age	30–39 years	50 (41.0)
	40–49 years	48 (39.3)
	50–59 years	24 (19.7)
Type of hospital	Designated rehabilitation hospital	58 (47.5)
	Tertiary hospital	40 (32.8)
	Rehabilitation hospital	11 (9.0)
	General hospital	10 (8.2)
	Clinic	3 (2.5)
Job title	Employed physicians	71 (58.2)
	Professor	40 (32.8)
	Self-employed physicians	9 (7.4)
	Fellowship	2 (1.6)
Years of board certification as physiatrist	0–10 years	67 (54.9)
	11–20 years	37 (30.3)
	Over 20 years	18 (14.8)

All data are presented with *n* (%).

## 3 Results

### 3.1 Demographics

Of the total 1,129 surveys sent out, 122 were returned [response rate: email (60/840), postal (62/289)]. The demographics of the respondents are presented in Table 1. The majority of respondents were male (72.1%), with a mean age of 42.2 years. The most prevalent workplace was “designated rehabilitation hospital” (47.5%), followed by “tertiary hospital” (32.8%). Most of the respondents were employed physicians (58.2%), followed by professors (32.8%). Among respondents, 45.1% had been working as a physiatrist for over 10 years.

### 3.2 Survey results of rTMS therapy

#### 3.2.1 Experience with rTMS and perspective of effectiveness

Among the 122 respondents, 92.62% (*n* = 113) had experience with rTMS for treatment of patients with stroke. Among those with experience, 6.6% reported that rTMS was “very effective” in the treatment of post-stroke impairments, 47.5% reported “effective,” 31.1% reported “somewhat effective,” and 14.8% reported “less effective” (Supplementary material 2). At the time of the survey, 69.7% (*n* = 85) of respondents were actively performing rTMS therapy for patients with stroke at their hospital. Among them, all respondents applied the rTMS for motor impairment, followed by language dysfunction (69.4%) and cognitive impairment (30.6%).

Regarding the question of whether the rTMS coil is securely anchored in the intended position during treatment, 3.5% reported “very stable,” 30.6% reported “stable,” 49.4% reported “moderately stable,” and 16.5% reported “very unstable” (Supplementary material 2). Further, 57.6% of respondents indicated that they fix the rTMS coil with an extra arm and adjust it along with the patient’s movement during treatment sessions, while 23.5% responded that they fix the rTMS coil at the beginning of treatment by an arm but do not adjust the coil along with the patient’s movement.

Motor-evoked potentials are measured by 57.6% of respondents to determine the motor threshold (Table 2). Conversely, 35.3% only visually observe muscle twitch response. When queried about their methods for identifying the motor hot spot, 54.1% stated they measured the motor evoked potentials, while 42.4% used anatomical landmarks and calculated the proportional distance based on the standard 10–20 electroencephalogram (EEG) system. Some respondents mentioned that they measure motor evoked potentials in clinical practice, while they use functional magnetic resonance imaging (fMRI) or EEG in research to identify motor hotspots.

**Table 2:** Clinical implementation status of rTMS for patients with stroke.

Item	Answer	Total (*n* = 85)
How do you determine the motor threshold?	Measurement of motor evoked potentials	49 (57.6)
	Visual observation of muscle twitch	30 (35.3)
	Not identifying motor threshold	6 (7.1)
How do you determine the motor hot spot?	Measurement of motor evoked potentials	46 (54.1)
	C3/C4 in the standard 10–20 system (EEG)	36 (42.4)
	Not identifying motor hot spot	2 (2.4)
What method do you apply to keep the coil in the initial stimulation target?	Fix the coil with an extra arm (O)	49 (57.6)
	Adjust the coil along with the patient’s movement (O)	
	Fix the coil with an extra arm and (O)	20 (23.5)
	Adjust the coil along with the patient’s movement (X)	
	Hold the coil manually (O)	14 (16.5)
	Adjust the coil along with the patient’s movement (O)	
	Hold the coil manually (O)	2 (2.4)
	Adjust the coil along with the patient’s movement (X)	

All data are presented with *n* (%). rTMS, Repetitive transcranial magnetic stimulation; EEG, Electroencephalogram.

#### 3.2.2 Physician awareness of rTMS

**Figure 1:**
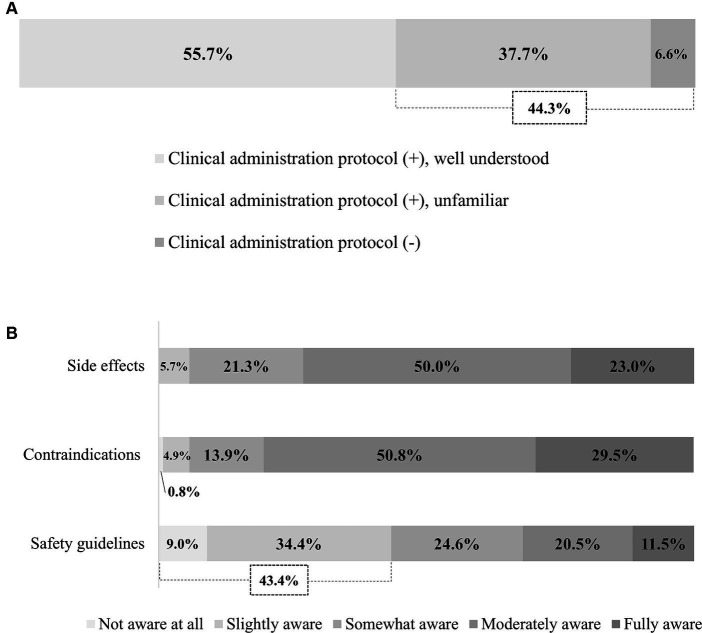
Physician awareness of rTMS. **(A)** Awareness of treatment protocol. **(B)** Awareness of side effects, contraindications, and safety guidelines. rTMS, Repetitive transcranial magnetic stimulation.

Survey responses indicated that 44.3% of hospitals either lack a treatment protocol, or where such a protocol exists, physiatrists are not familiar with it (Figure 1A). Most respondents were well aware of the side effects (e.g., headache, hearing problem, and seizure) and contraindications (e.g., the presence of metal hardware, history of seizures, and mental illness) of rTMS. However, 43.4% were relatively unaware of safety guidelines (e.g., total number of pulses, stimulation intensity) (Figure 1B). The most concerning side effects were seizure (97.5%), followed by headache (36.9%) (Supplementary material 2).

#### 3.2.3 Unmet needs for rTMS therapy in stroke rehabilitation

**Figure 2:**
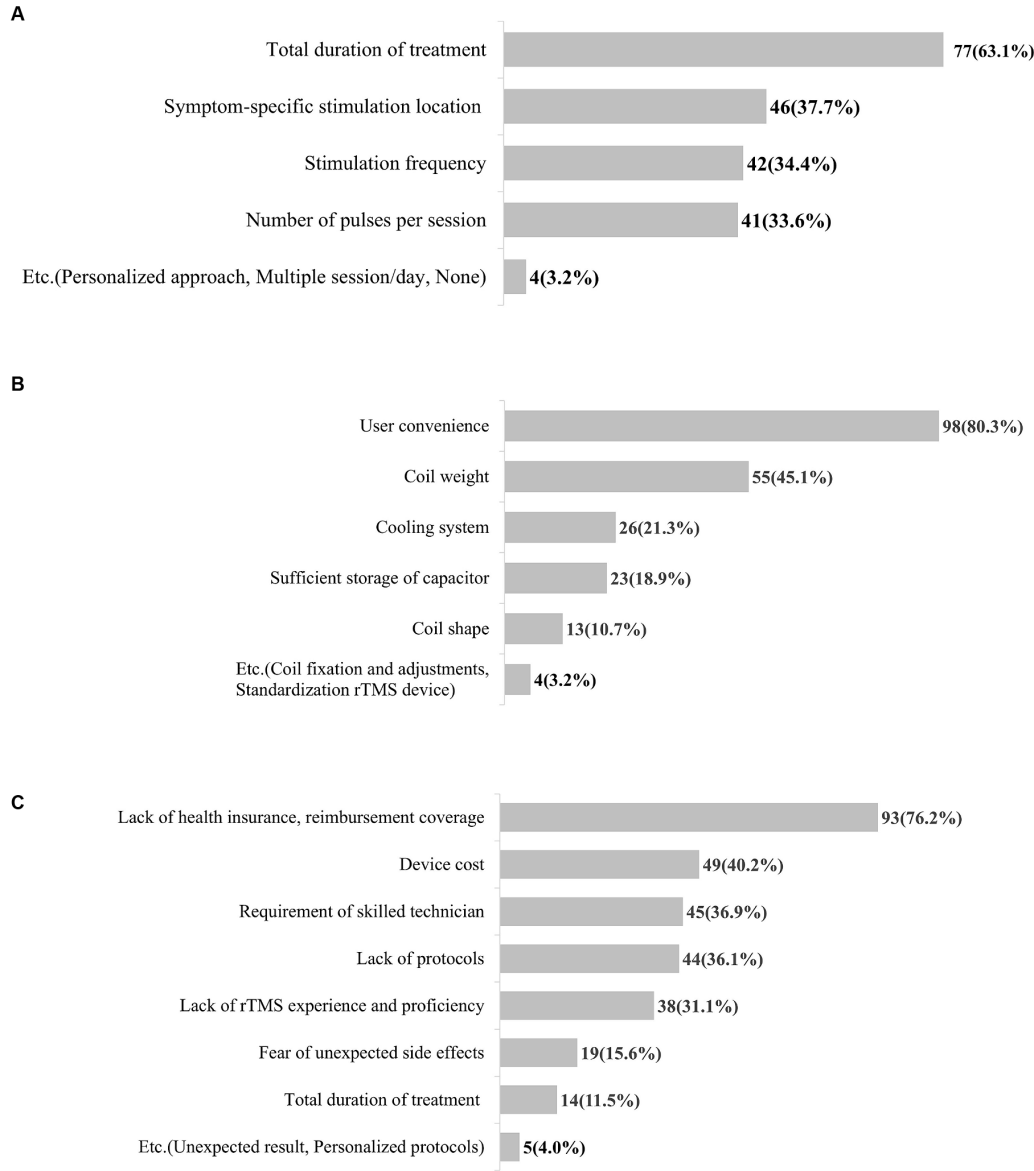
Unmet needs for rTMS therapy in patients with stroke. **(A)** Unmet needs for treatment protocol. **(B)** Unmet needs for rTMS device. **(C)** Barriers to clinical application of rTMS therapy. rTMS, Repetitive transcranial magnetic stimulation.

Respondents had numerous unmet needs for rTMS therapy for treating patients with stroke. The total duration of treatment (1 week, 2 weeks, 4 weeks, or other) was identified as the most difficult parameter when determining an rTMS therapy protocol for 63.1% of respondents, followed by the symptom-specific stimulation location (37.7%) (Figure 2A). Other responses included the consideration of a personalized approach and number of treatment sessions per day. Importantly, 80.3% of respondents wanted to enhance the usability of rTMS devices, followed by the need for lighter rTMS coils (45.1%) (Figure 2B). Of the respondents, 76.2% reported that the most significant barriers to the clinical application of rTMS are lack of health insurance and reimbursement coverage followed by device cost, requirement of a skilled technician, and protocols (Figure 2C). We obtained additional open-ended responses regarding unmet needs for rTMS therapy and categorized them: the most common unmet needs were lack of protocols, guidelines, and education (30.6%), followed by usability of rTMS devices (28.6%) (Supplementary material 2).

### 3.3 Survey results of neuro-navigation system

#### 3.3.1 Experience with and perspective of neuro-navigation system

**Figure 3:**
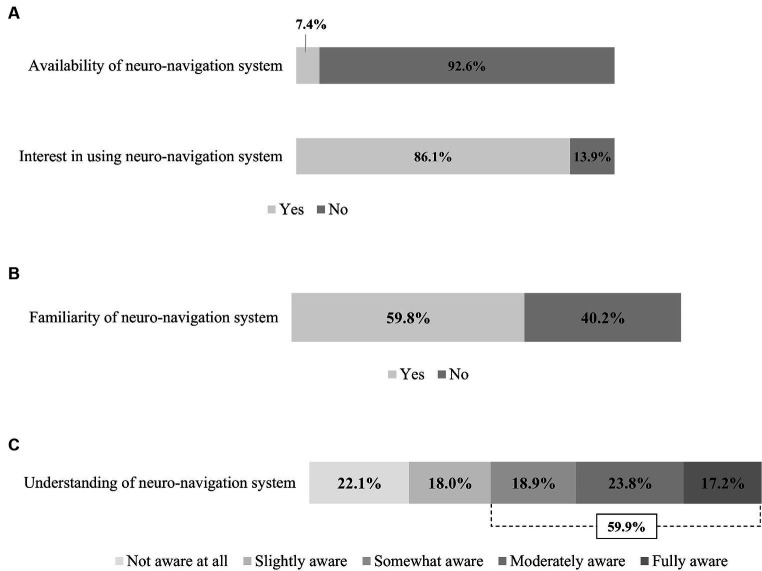
Usage and physician awareness of the neuro-navigation system. **(A)** Availability and interest of neuro-navigation system. **(B)** Familiarity with neuro-navigation system. **(C)** Understanding of neuro-navigation system.

Only 7.4% of respondents indicated that their institution has a neuro-navigation system (Figure 3A). However, 86.1% responded that they would be willing to use a neuro-navigation system for rTMS therapy if it were available.

#### 3.3.2 Physician awareness of neuro-navigation systems

Of all respondents, 59.8% reported that they had heard of or were familiar with neuro-navigation (Figure 3B). Correspondingly, 59.9% of the respondents reported that they were aware of the concept of using neuro-navigation in rTMS therapy (Figure 3C).

#### 3.3.3 Unmet needs of neuro-navigation system

**Figure 4:**
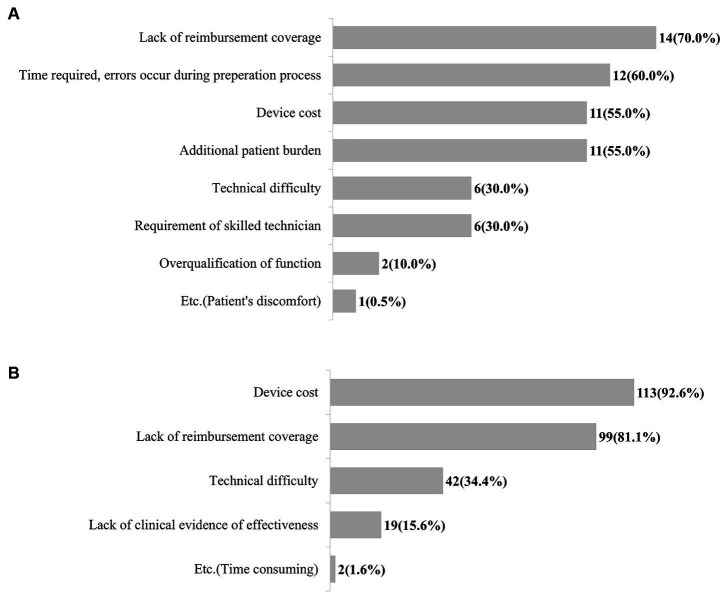
Unmet needs for neuro-navigation system. **(A)** Shortcomings and limitations of commercialized neuro-navigation systems. **(B)** Barriers to clinical application of neuro-navigation system.

Among the respondents currently using neuro-navigation systems, 70% reported a lack of reimbursement coverage of prescription as a major limitation for usage, followed by the time required, errors occurring in the preparation process, the device cost, and the additional burden on the patient [e.g., requirement of navigation magnetic resonance imaging (MRI)] (Figure 4A). The most common barrier to the application of neuro-navigation systems in clinical practice was the cost of the device (92.6%), followed by lack of reimbursement coverage (Figure 4B).

## 4 Discussion

Repetitive transcranial magnetic stimulation is widely used in clinical practice and research for stroke rehabilitation. The primary objective of this study was to assess the current status and identify unmet clinical needs related to rTMS treatment and neuro-navigation in stroke rehabilitation. The results of our nationwide survey indicated that most physiatrists are familiar with rTMS and agree with its effectiveness, especially for motor impairment after stroke. rTMS itself is not a novel treatment; however, numerous unmet needs remain, including a standardized treatment protocol, optimal patient selection criteria, adjuvant application techniques, and reimbursement coverage. In addition, unmet needs for neuro-navigation systems also exist. These systems may accelerate the accuracy and reliability of rTMS; nonetheless, they are not well known among clinicians yet, and more scalable devices are needed.

Repetitive transcranial magnetic stimulation has the potential to alter neural plasticity by inducing depolarization in neural cells at the cortex level through the electric currents generated by magnetic stimulation. Furthermore, it modulates the functional connectivity between these neurons and adjacent neural substrates (24). It is a relatively safe, non-invasive brain stimulation technique that has been studied and used in clinical practice in various disease entities.

However, despite the increasing evidence, rTMS is not generally recommended by experts in stroke rehabilitation (25). One reason is that, although several studies have demonstrated the clinical efficacy of rTMS in stroke patients (26–28), there remains a lack of consensus on the potential therapeutic effect of rTMS (16).

Our results revealed that the perceived efficacy of rTMS in stroke recovery was not as high as expected (moderately or highly effective: 54.1%). Individual randomized clinical trials (RCTs) have shown positive results on the effectiveness of rTMS for patients with stroke (25, 29). However, according to a Cochrane review, Hao et al. (16) found that it is difficult to draw a clear conclusion on the effectiveness of rTMS in post-stroke patients, and Pollock et al. (30) found that rTMS was effective in improving upper limb function and performance of activities of daily living tasks; however, the level of evidence was low. Heterogenous effect sizes between studies, small sample sizes, and various proof-of-concept trials have led to uncertain evidence in a previous study (25). In contrast, a 2017 meta-analysis by Zhang et al. (7) reported short- and long-term treatment effects on upper limb function recovery after stroke, indicating there is still no clear consensus on the treatment effects of rTMS in stroke rehabilitation.

To enhance the treatment efficacy of rTMS, it is necessary to provide accurate treatment protocols and appropriate guidelines (31). Our findings revealed only 37.7% of respondents have their own treatment protocol, yet are unfamiliar with it, and 6.6% have no protocol whatsoever. Further, 30.6% of respondents have unmet needs for standardized clinical protocols and training programs for physicians and researchers. They also reported many challenges in determining the appropriate protocol according to the time since stroke, lesion location, severity, and total treatment period. As such, the lack of standardized treatment protocols is a barrier to the active use of rTMS in clinical practice. Similarly, Fisicaro et al. (9) demonstrated there is considerable variation across studies in stimulus frequency, intensity, number of sessions, number of total pulses, and total treatment duration. Additionally, there is no consensus on these parameters, to date.

The effectiveness of rTMS is highly dependent on both the selection of the stimulation target and the precision of the methods used to find this target (32). Especially, when applying rTMS to post-stroke impairment, accurately identifying the “motor hotspot” and determining “motor threshold” are essential. The motor hotspot is the location at the motor cortex where a stimulus of a given intensity produces maximum peak-to-peak motor-evoked potential (MEP) (33, 34). In our survey, 57.6% of respondents reported that they determine the motor threshold by evaluating the MEP; however, 35.3% only observe the muscle twitch. The latter method tends to overestimate the motor threshold by 11.3%, which may be a safety concern for some patients or in certain protocols (35). Approximately 43% of respondents reported that they use the 10–20 EEG system to identify “motor hot spots,” rather than MEP measurement, which may also hinder the treatment effect because it is not an accurate stimulation target based on a functional approach.

Other important features of increasing the effectiveness of rTMS therapy are accuracy and repeatability of coil application. Maintaining the position of the rTMS coil is crucial during the treatment because the induced magnetic field at the cortex can be significantly changed by even small movements in orientation of the rTMS coil (36). In our study, only 34.1% (highly stable: 3.5%, stable: 30.6%) of respondents were confident that the magnetic coil remains in its originally intended position during rTMS therapy. rTMS therapy often lasts 5–25 min per session and is repeated over several days to weeks, during which magnetic stimulation should be applied to the same location. This highlights the need for a system that ensures the coil position in real time. Moreover, the shape and size of the brain and head, distance between the stimulation coil and the responding neural tissue, and orientation and location of anatomic structures are all parameters that must be defined for each patient in rTMS therapy (37).

Neuro-navigation is useful in this context to identify the stimulation target based on MRI and to track the coil in real time. It is able to correct any movement error in position or angulation of the rTMS coil during the session (38), and it also provides optimal reproducibility between sessions (39). Fitzgerald et al. (40) reported that navigation rTMS, compared to standard rTMS, enabled more precise targeting of specific areas for the treatment of depression, resulting in improved outcomes. Despite the many advantages of neuro-navigation (41), it is rarely used in clinical practice for TMS therapy (42). In our survey, 40% of respondents reported they had never heard of neuro-navigation systems or did not know about its concept; only 7.4% of respondents were currently using neuro-navigation in rTMS therapy.

While neuro-navigation can improve the accuracy and precision of rTMS therapy, it has some major limitations. First, numerous technical and man-made errors occur unexpectedly during procedures. The neuro-navigation process is typically classified into computed tomography (CT)- or MRI-based image acquisition, planning, patient-to-image registration, and mechanical execution phases. The largest errors may occur in the surface registration phase, and the accumulation of errors in each phase increases the probability of applying magnetic stimulation to unintended brain cortex areas (43). Users of neuro-navigation systems may have a misconception that using neuro-navigation will definitely lead to superior outcomes; however, clinicians should be aware of the various errors that may occur.

According to our survey, unmet needs for rTMS devices included usability improvements, followed by lighter weight of the magnetic coil. In addition, barriers to clinical adoption of rTMS systems included a lack of health insurance and reimbursement coverage, as well as device cost. The current rTMS systems are heavy, complicated, and expensive, which can be a barrier to the use of rTMS in small clinic applications (44). Consistent with our findings, Pacheco-Barrios et al. (45) reported that addressing potential barriers to device usability, such as lightening the weight, improving scalp comfort, and avoiding excessive heat, also increases device adoption.

In addition, in our survey, unmet needs for neuro-navigation systems included appropriate reimbursement coverage, followed by device costs, technical barriers and errors, and additional burden on patients and physicians. Some commercialized neuro-navigation systems are overengineered and not suitable for rTMS, preventing its wide usage. Previous studies also reported that neuro-navigated rTMS therapy adds complexity and additional cost, and has yet to be definitively demonstrated to improve clinical outcomes (46). Moreover, Caulfield et al. (41) noted that proficient use of neuro-navigation in rTMS requires a streamlined approach and additional training for technicians, including an understanding of the software and camera interface, for accurate application. Notably, 86.1% of respondents in our survey expressed interest in a neuro-navigation system if available. Therefore, if an easy-to-use and advanced neuro-navigation system specialized for rTMS is developed; it will not only be widely used in clinical practice but will also contribute to improving the quality of rTMS-related research.

A few limitations of the present study warrant acknowledgment. First, despite the nationwide scope of the survey, its target was solely physiatrists, potentially leading to an underrepresentation of all clinicians who might use this treatment method. However, considering that in South Korea the majority of post-stroke rTMS therapies are administered by departments of rehabilitation, we assumed the survey effectively captured substantial insights into the unmet needs within this domain. However, future research should aim for a more representative sample that includes diverse hospital environments and working modes to enhance the generalizability of the findings. Second, the sample size was limited, with a response rate of approximately 10.8%. Despite the survey being conducted using email and postal service, the data set generated was insufficient for creating a comprehensive and representative sample. However, it is important to note that medical professionals applying rTMS in stroke rehabilitation are concentrated in specific groups. In this study, approximately 70% of the respondents reported actively using rTMS in their clinical practice. This suggests that the data collected can be considered somewhat representative of the current state of rTMS utilization in stroke rehabilitation. Third, no correlation analyses were performed. We focused on descriptive statistics to align with the exploratory nature of our survey, which aimed to gather foundational data. In future studies, a more systematic approach to designing the survey questionnaire may enable correlation analysis, potentially yielding valuable insights into the relationships between various factors influencing rTMS utilization in stroke rehabilitation. Lastly, patient factors such as awareness and acceptance of rTMS were not assessed, yet they significantly impact its application. Future research should include evaluations of these patient-related factors to provide a more comprehensive understanding of the barriers to rTMS utilization. This approach will assist in the development of effective strategies to enhance patient education and acceptance, thereby facilitating wider adoption of rTMS in clinical practice.

## 5 Conclusion

Although widely acknowledged for its effectiveness, particularly in motor impairment rehabilitation, rTMS faces challenges in clinical adoption due to a lack of standardized treatment protocols, sufficient evidence for its efficacy, and issues related to device usability and insurance coverage in stroke rehabilitation. The findings from our nationwide survey of physiatrists indicated a demand for clearer guidelines, better education on rTMS application, and the development of more accessible neuro-navigation systems to enhance treatment precision. Addressing these unmet needs is crucial for bridging the gap between research and clinical practice, thereby maximizing the therapeutic benefits of rTMS in stroke rehabilitation.

## Data availability statement

The original contributions presented in the study are included in the article/Supplementary material; further inquiries can be directed to the corresponding author.

## Author contributions

GY: Conceptualization, Formal analysis, Investigation, Visualization, Writing – original draft, Writing – review & editing. CP: Data curation, Investigation, Writing – original draft. MJ: Data curation, Investigation, Writing – original draft. GJ: Data curation, Investigation, Writing – original draft. KK: Conceptualization, Supervision, Project administration, Methodology, Writing – review & editing.

## References

[1] Motolese F Capone F Di Lazzaro V. New tools for shaping plasticity to enhance recovery after stroke. Handb Clin Neurol. (2022) 184:299–315. doi: 10.1016/B978-0-12-819410-2.00016-3, PMID: 35034743

[2] Langhorne P Coupar F Pollock A. Motor recovery after stroke: a systematic review. Lancet Neurol. (2009) 8:741–54. doi: 10.1016/S1474-4422(09)70150-4 19608100

[3] Mally J Dinya E. Recovery of motor disability and spasticity in post-stroke after repetitive transcranial magnetic stimulation (rTMS). Brain Res Bull. (2008) 76:388–95. doi: 10.1016/j.brainresbull.2007.11.019, PMID: 18502315

[4] Buma F Kwakkel G Ramsey N. Understanding upper limb recovery after stroke. Restor Neurol Neurosci. (2013) 31:707–22. doi: 10.3233/RNN-130332 23963341

[5] Ridding MC Rothwell JC. Perspectives—opinion—is there a future for therapeutic use of transcranial magnetic stimulation? Nat Rev Neurosci. (2007) 8:559–67. doi: 10.1038/nrn2169, PMID: 17565358

[6] Rossi S Hallett M Rossini PM Pascual-Leone A Safety of TMSCG. Safety, ethical considerations, and application guidelines for the use of transcranial magnetic stimulation in clinical practice and research. Clin Neurophysiol. (2009) 120:2008–39. doi: 10.1016/j.clinph.2009.08.016, PMID: 19833552 PMC3260536

[7] Zhang L Xing G Fan Y Guo Z Chen H Mu Q. Short- and long-term effects of repetitive transcranial magnetic stimulation on upper limb motor function after stroke: a systematic review and meta-analysis. Clin Rehabil. (2017) 31:1137–53. doi: 10.1177/0269215517692386 28786336

[8] Xiang H Sun J Tang X Zeng K Wu X. The effect and optimal parameters of repetitive transcranial magnetic stimulation on motor recovery in stroke patients: a systematic review and meta-analysis of randomized controlled trials. Clin Rehabil. (2019) 33:847–64. doi: 10.1177/0269215519829897, PMID: 30773896

[9] Fisicaro F Lanza G Grasso AA Pennisi G Bella R Paulus W. Repetitive transcranial magnetic stimulation in stroke rehabilitation: review of the current evidence and pitfalls. Ther Adv Neurol Disord. (2019) 12:175628641987831. doi: 10.1177/1756286419878317 PMC6763938 31598137

[10] Otal B Olma MC Flöel A Wellwood I. Inhibitory non-invasive brain stimulation to homologous language regions as an adjunct to speech and language therapy in post-stroke aphasia: a meta-analysis. Front Hum Neurosci. (2015) 9:236. doi: 10.3389/fnhum.2015.00236, PMID: 25972805 PMC4412051

[11] Ren C Zhang G Xu X Hao J Fang H Chen P. The effect of rTMS over the different targets on language recovery in stroke patients with global aphasia: a randomized sham-controlled study. Biomed Res Int. (2019) 2019:4589056–7. doi: 10.1155/2019/4589056, PMID: 31467892 PMC6699349

[12] Hu XY Zhang T Rajah GB Stone C Liu LX He JJ. Effects of different frequencies of repetitive transcranial magnetic stimulation in stroke patients with non-fluent aphasia: a randomized, sham-controlled study. Neurol Res. (2018) 40:459–65. doi: 10.1080/01616412.2018.1453980, PMID: 29589518

[13] Georgiou AM Lada E Kambanaros M. Evaluating the quality of conduct of systematic reviews on the application of transcranial magnetic stimulation (TMS) for aphasia rehabilitation post-stroke. Aphasiology. (2020) 34:540–56. doi: 10.1080/02687038.2019.1632786

[14] Hsiao MY Choo YJ Liu IC Boudier-Reveret M Chang MC. Effect of repetitive transcranial magnetic stimulation on post-stroke dysphagia: a Meta-analysis of stimulation frequency, stimulation site, and timing of outcome measurement. Dysphagia. (2023) 38:435–45. doi: 10.1007/s00455-022-10483-9, PMID: 35763122

[15] Georgiou AM Phylactou P Kambanaros M. The effectiveness of transcranial magnetic stimulation for dysphagia in stroke patients: an umbrella review of systematic reviews and meta-analyses. Front Hum Neurosci. (2024) 18:1355407. doi: 10.3389/fnhum.2024.1355407, PMID: 38550720 PMC10972992

[16] Hao Z Wang D Zeng Y Liu M. Repetitive transcranial magnetic stimulation for improving function after stroke. Cochrane Database Syst Rev. (2013) 2013:CD008862. doi: 10.1002/14651858.CD008862.pub2, PMID: 23728683 PMC6464739

[17] Chino T Kinoshita S Abo M. Repetitive transcranial magnetic stimulation and rehabilitation therapy for upper limb hemiparesis in stroke patients: a narrative review. Prog Rehabil Med. (2023) 8:20230005. doi: 10.2490/prm.20230005, PMID: 36866154 PMC9970844

[18] Zemplényi A Józwiak-Hagymásy J Kovács S Erdosi D Boncz I Tényi T. Repetitive transcranial magnetic stimulation may be a cost-effective alternative to antidepressant therapy after two treatment failures in patients with major depressive disorder. BMC Psychiatry. (2022) 22:22. doi: 10.1186/s12888-022-04078-9, PMID: 35764989 PMC9238085

[19] Laufer J Olmsted A Sampair I Madore M Yoon J Hack L. Sequential acute courses of transcranial magnetic stimulation in major depressive disorder: a retrospective analysis in a veteran cohort. J Affect Disord Rep. (2024) 17:100801. doi: 10.1016/j.jadr.2024.100801

[20] Baeken C Arns M Brunelin J Chanes L Filipcic I Ganho-Avila A. European reclassification of non-invasive brain stimulation as class III medical devices: a call to action. Brain Stimul. (2023) 16:564–6. doi: 10.1016/j.brs.2023.02.012, PMID: 36870602 PMC7618788

[21] Chipchase L Schabrun S Cohen L Hodges P Ridding M Rothwell J. A checklist for assessing the methodological quality of studies using transcranial magnetic stimulation to study the motor system: an international consensus study. Clin Neurophysiol. (2012) 123:1698–704. doi: 10.1016/j.clinph.2012.05.003, PMID: 22647458 PMC4884647

[22] Eysenbach G. Improving the quality of web surveys: The checklist for reporting results of internet E-surveys (CHERRIES). J Med Internet Res. (2004) 6:e34. doi: 10.2196/jmir.6.3.e34 15471760 PMC1550605

[23] Lefaucheur JP Aleman A Baeken C Benninger DH Brunelin J Di Lazzaro V. Evidence-based guidelines on the therapeutic use of repetitive transcranial magnetic stimulation (rTMS): an update (2014-2018). Clin Neurophysiol. (2020) 131:474–528. doi: 10.1016/j.clinph.2019.11.002, PMID: 31901449

[24] Kricheldorff J Göke K Kiebs M Kasten FH Herrmann CS Witt K. Evidence of Neuroplastic changes after transcranial magnetic, electric, and deep brain stimulation. Brain Sci. (2022) 12:929. doi: 10.3390/brainsci12070929, PMID: 35884734 PMC9313265

[25] Hofmeijer J Ham F Kwakkel G. Evidence of rTMS for motor or cognitive stroke recovery: hype or Hope? Stroke. (2023) 54:2500–11. doi: 10.1161/STROKEAHA.123.043159 37747964

[26] Ameli M Grefkes C Kemper F Riegg FP Rehme AK Karbe H. Differential effects of high-frequency repetitive transcranial magnetic stimulation over ipsilesional primary motor cortex in cortical and subcortical middle cerebral artery stroke. Ann Neurol. (2009) 66:298–309. doi: 10.1002/ana.21725, PMID: 19798637

[27] Khedr EM Abdel-Fadeil MR Farghali A Qaid M. Role of 1 and 3 Hz repetitive transcranial magnetic stimulation on motor function recovery after acute ischaemic stroke. Eur J Neurol. (2009) 16:1323–30. doi: 10.1111/j.1468-1331.2009.02746.x, PMID: 19780802

[28] Yozbatiran N Alonso-Alonso M See J Demirtas-Tatlidede A Luu D Motiwala RR. Safety and behavioral effects of high-frequency repetitive transcranial magnetic stimulation in stroke. Stroke. (2009) 40:309–12. doi: 10.1161/STROKEAHA.108.522144, PMID: 18845801 PMC3366156

[29] Sheng R Chen C Chen H Yu P. Repetitive transcranial magnetic stimulation for stroke rehabilitation: insights into the molecular and cellular mechanisms of neuroinflammation. Front Immunol. (2023) 14:1197422. doi: 10.3389/fimmu.2023.1197422, PMID: 37283739 PMC10239808

[30] Pollock A Baer G Campbell P Choo PL Forster A Morris J. Physical rehabilitation approaches for the recovery of function and mobility following stroke. Cochrane Database Syst Rev. (2014) 2023:CD001920. doi: 10.1002/14651858.CD001920.pub3, PMID: 24756870 PMC6465059

[31] Adeyemo BO Simis M Macea DD Fregni F. Systematic review of parameters of stimulation, clinical trial design characteristics, and motor outcomes in non-invasive brain stimulation in stroke. Front Psychol. (2012) 3:88. doi: 10.3389/fpsyt.2012.00088, PMID: 23162477 PMC3495265

[32] Herbsman T Avery D Ramsey D Holtzheimer P Wadjik C Hardaway F. More lateral and anterior prefrontal coil location is associated with better repetitive transcranial magnetic stimulation antidepressant response. Biol Psychiatry. (2009) 66:509–15. doi: 10.1016/j.biopsych.2009.04.034 19545855

[33] Rothwell JC Hallett M Berardelli A Eisen A Rossini P Paulus W. Magnetic stimulation: motor evoked potentials. The International Federation of Clinical Neurophysiology. Electroencephalogr Clin Neurophysiol Suppl. (1999) 52:97–103. PMID: 10590980

[34] Rossini PM Burke D Chen R Cohen LG Daskalakis Z Di Iorio R. Non-invasive electrical and magnetic stimulation of the brain, spinal cord, roots and peripheral nerves: basic principles and procedures for routine clinical and research application. An updated report from an I.F.C.N. Committee. Clin Neurophysiol. (2015) 126:1071–107. doi: 10.1016/j.clinph.2015.02.001, PMID: 25797650 PMC6350257

[35] Westin GG Bassi BD Lisanby SH Luber B Inst NYSP. Determination of motor threshold using visual observation overestimates transcranial magnetic stimulation dosage: safety implications. Clin Neurophysiol. (2014) 125:142–7. doi: 10.1016/j.clinph.2013.06.187, PMID: 23993680 PMC3954153

[36] Danner N Julkunen P Kononen M Saisanen L Nurkkala J Karhu J. Navigated transcranial magnetic stimulation and computed electric field strength reduce stimulator-dependent differences in the motor threshold. J Neurosci Methods. (2008) 174:116–22. doi: 10.1016/j.jneumeth.2008.06.032, PMID: 18662721

[37] Ruohonen J Karhu J. Navigated transcranial magnetic stimulation. Neurophysiol Clin Clin Neurophysiol. (2010) 40:7–17. doi: 10.1016/j.neucli.2010.01.006 20230931

[38] Schonfeldt-Lecuona C Lefaucheur JP Cardenas-Morales L Wolf RC Kammer T Herwig U. The value of neuronavigated rTMS for the treatment of depression. Neurophysiol Clin. (2010) 40:37–43. doi: 10.1016/j.neucli.2009.06.004, PMID: 20230934

[39] Lefaucheur JP. Why image-guided navigation becomes essential in the practice of transcranial magnetic stimulation. Neurophysiol Clin. (2010) 40:1–5. doi: 10.1016/j.neucli.2009.10.004, PMID: 20230930

[40] Fitzgerald PB Hoy K McQueen S Maller JJ Herring S Segrave R. A randomized trial of rTMS targeted with MRI based neuro-navigation in treatment-resistant depression. Neuropsychopharmacology. (2009) 34:1255–62. doi: 10.1038/npp.2008.233, PMID: 19145228

[41] Caulfield KA Fleischmann HH Cox CE Wolf JP George MS McTeague LM. Neuronavigation maximizes accuracy and precision in TMS positioning: evidence from 11,230 distance, angle, and electric field modeling measurements. Brain Stimul. (2022) 15:1192–205. doi: 10.1016/j.brs.2022.08.013, PMID: 36031059 PMC10026380

[42] Wang H Cui D Jin J Wang X Li Y Liu Z. 3D-printed helmet-type neuro-navigation approach (I-helmet) for transcranial magnetic stimulation. Front Neurosci. (2023) 17:1224800. doi: 10.3389/fnins.2023.1224800, PMID: 37609452 PMC10442160

[43] Batista PD Machado IP Roios P Lavrador J Cattoni MB Martins J. Position and orientation errors in a Neuronavigation procedure: a stepwise protocol using a cranial phantom. World Neurosurg. (2019) 126:E342–50. doi: 10.1016/j.wneu.2019.02.052, PMID: 30822590

[44] Health Quality Ontario. Repetitive transcranial magnetic stimulation for treatment-resistant depression: an economic analysis. Ont Health Technol Assess Ser. (2016) 16:1–51. PMC4808718 27110317

[45] Pacheco-Barrios K Gianlorenco AC Camargo L Dodurgali MR Tangjade A Fregni F. Accelerating the development of noninvasive brain stimulation devices: using design thinking to facilitate its clinical use and acceptance. Expert Rev Neurother. (2024) 24:5–9. doi: 10.1080/14737175.2023.2292733, PMID: 38149610 PMC10983014

[46] Fitzgerald PB. Targeting repetitive transcranial magnetic stimulation in depression: do we really know what we are stimulating and how best to do it? Brain Stimul. (2021) 14:730–6. doi: 10.1016/j.brs.2021.04.018, PMID: 33940242

