# Supplementary material for: Clinical implementation, barriers, and unmet needs of rTMS and neuro-navigation systems in stroke rehabilitation: a nationwide survey in South Korea
Source: Front Neurol. 2024 Jul 30;15:1423013. doi: 10.3389/fneur.2024.1423013 (PMC11321079; doi:10.3389/fneur.2024.1423013)
Supplement: Supplementary file 1 [file Data_Sheet_1.DOCX]

Supplementary Material

# Supplementary Material 1. Questionnaire

**Section 1. Baseline demographics**

01. Please enter your email address.

:______________________________

02. What is your gender?

□ Male □ Female

03. What is your age?

:_____________

04. What's your mobile phone number (used only for reward)?

: ______________________________

05. What is your medical license number?

: ______________________________

06. What is the type of hospital you work in?

A. Tertiary Hospital

B. General Hospital

C. Designated rehabilitation hospital

D. Rehabilitation hospital (excluding designated rehabilitation hospital)

E. Clinic

07. Which of the following job titles best describes your current position?

A. Professor

B. Employed physicians

C. Self-employed physicians

D. Fellowship

E. Public health doctor

08. What year did you become a rehabilitation specialist (Physiatrist)?

: _________________

**Section 2. The following are questions about rTMS treatment.**

01. Have you ever applied the rTMS for patients with stroke, and if so, for how long?

A. None

B. Less than 1 year

C. 1-5 years

D. More than 5 years

02. Do you think rTMS is effective for patient with stroke?

(1= Not effective at all; 2 = Slightly effective; 3 = Somewhat effective; 4 = Moderately effective; 5 = Highly effective)

|  | 1 | 2 | 3 | 4 | 5 |  |
| --- | --- | --- | --- | --- | --- | --- |
| Not effective at all | ○ | ○ | ○ | ○ | ○ | Highly effectiveVery |

03. Are you currently appling rTMS for patients with stroke?

A. Yes.

B. No. (Go to question 9)

04. How do you determine the motor threshold and stimulation intensity for rTMS treatment?

*motor threshold: The minimum magnetic stimulation intensity required to produce a motor-evoked potential.

A. Determine the therapeutic magnetic stimulation intensity without identifying motor threshold .

B. Visual observation of muscle twitch.

C. Measurement of motor evoked potentials.

D. Other: _________________

05. How do you determine the motor hot spot?

*Motor hot spot: The area of the motor cortex that, when stimulated, produces the maximum amplitude, shortest latency, and most reliable motor-evoked potential (MEP) in a specific muscle.

A. Not identifying motor hot spot.

B. C3/C4 in the standard 10-20 system for electroencephalogram (EEG).

C. Measurement of motor evoked potentials.

D. Measurement of EEG.

E. Measurement of functional Magnetic Resonance Imaging (fMRI).

F. Other :_______________

06. During an rTMS treatment, what method do you apply to keep the coil in the initial stimulation target?

A. Hold the coil manually and do not adjust the coil with the patient's movements.

B. Hold the coil manually and adjust the coil with the patient's movements.

C. Fix the coil with an extra arm and do not adjust the coil along with the patient's movement.

D. Fix the coil with an extra arm and adjust the coil along with the patient's movement.

07. Do you think the magnetic coil is maintained in the initial intended location for the duration of the rTMS treatment?

(1 =Very Unstable; 2 = Somewhat Unstable; 3 = Moderately Stable; 4 = Stable; 5 = Very Stable)

|  | 1 | 2 | 3 | 4 | 5 |  |
| --- | --- | --- | --- | --- | --- | --- |
| Very Unstable | ○ | ○ | ○ | ○ | ○ | Very Stable |

08. For which symptoms do you apply the rTMS for stroke patients? (Select up to 3)

A. Motor dysfunction

B. Language dysfunction

C. Dysphagia

D. Hemispatial neglect

E. Depression

F. Cognitive impairment

G. Central neuropathic pain

H. Spasticity

09. For which symptoms do you think rTMS is most effective for stroke patients? (Select up to 3)

A. Motor dysfunction

B. Language dysfunction

C. Dysphagia

D. Hemispatial neglect

E. Depression

F. Cognitive impairment

G. Central neuropathic pain

H. Spasticity

10. Does your have a protocol for rTMS in patients with stroke and are you familiar with it?

A. I have treatment protocol and I am well aware of it.

B. I have treatment protocol but I am unfamiliar with it.

C. I don’t have a treatment protocol.

11. Which of the following parameters are difficult to determine in an rTMS treatment protocol? (Select up to 3)

A. Symptom-specific stimulation location (M1, DLPFC, Parietal lobe, etc.)

B. Stimulation frequency [low frequency (1 Hz), high frequency (10 Hz), theta wave (50 Hz)].

C. Number of pulses per session (300, 600, etc.)

D. Total duration of treatment (1 week, 2 weeks, 4 weeks, etc.)

E. Other : ______________________________

12. What are some other important considerations in rTMS protocols (e.g., disease severity, brain lesion, time since stroke onset, etc.)?

: ___________________________

13. Do you know the major side effects of rTMS?

(1 = Not aware at all; 2 = Slightly aware; 3 = Somewhat aware; 4 = Moderately aware; 5 = Fully aware)

|  | 1 | 2 | 3 | 4 | 5 |  |
| --- | --- | --- | --- | --- | --- | --- |
| Not aware at all | ○ | ○ | ○ | ○ | ○ | Fully aware |

14. Which side effects are you most concerned about when applying rTMS in patients with stroke? (Select up to 3)

A. Pain at the stimulation site

B. Seizure

C. Headache

D. Hearing problem

E. Other : ______________________________

15. Do you know contraindications to rTMS in patients with stroke?

(1 = Not aware at all; 2 = Slightly aware; 3 = Somewhat aware; 4 = Moderately aware; 5 = Fully aware)

|  | 1 | 2 | 3 | 4 | 5 |  |
| --- | --- | --- | --- | --- | --- | --- |
| Not aware at all | ○ | ○ | ○ | ○ | ○ | Fully aware |

16. Do you know the safety guidelines for rTMS (e.g. the maximum number of pulses per day)? (1 = Not aware at all; 2 = Slightly aware; 3 = Somewhat aware; 4 = Moderately aware; 5 = Fully aware)

|  | 1 | 2 | 3 | 4 | 5 |  |
| --- | --- | --- | --- | --- | --- | --- |
| Not aware at all | ○ | ○ | ○ | ○ | ○ | Fully aware |

17. What are the most significant barriers to the clinical application of rTMS for patients? (Select up to 3)

A. Lack of health insurance, reimbursement coverage

B. Lack of protocols

C. Device cost

D. Lack of rTMS experience and proficiency.

E. Requirement of skilled technician

F. Fear of unexpected side effects

G. Total duration of treatment

H. Other : ______________________________

18. What is the most important consideration to increase the therapeutic effectiveness of rTMS? (Select up to 3)

A. Appropriate patient selection

B. Optimal protocol

C. Appropriate localization of the stimulation target

D. Maintain the coil position during treatment

E. Appropriate treatment time since stroke onset

F. Other : ______________________________

19. What needs to be improved about the rTMS device? (Select up to 3)

A. User convenience (adjustment knobs, etc.)

B. Coil weight

C. Coil shape

D. Cooling system

E. Sufficient storage of capacitor (problem of decreasing magnetic field strength in theta-wave stimulation)

F. Other : ______________________________

20. What do you think is an acceptable budget for an rTMS device? (₩, WON)

A. ~10 million

B. 10 million ~ 50 million

C. 50 million ~ 100 million

D. More than 100 million

E. Other : ______________________________

**Section 3. The following are questions about Neuro-navigation systems in rTMS treatment**

01. Are you familiar with the application of neuro-navigation in rTMS treatment?

A. Yes.

B. No.

02. Do you know the concept and mechanism of a neuro-navigation system for rTMS treatment?

(1 = Not aware at all; 2 = Slightly aware; 3 = Somewhat aware; 4 = Moderately aware; 5 = Fully aware)

|  | 1 | 2 | 3 | 4 | 5 |  |
| --- | --- | --- | --- | --- | --- | --- |
| Not aware at all | ○ | ○ | ○ | ○ | ○ | Fully aware |

03. Does your hospital use a neuro-navigation during rTMS treatment?

A. Yes

B. No

04. Which of the following do you think is the reason for the limited use of neuro-navigation system in rTMS treatment? (select up to 4)

A. Technical difficulty

B. Device cost

C. Requirement of a skilled technician

D. Time requirement, errors occur during preparation process

E. Additional patient burden (e.g., navigation MRI)

F. Lack of reimbursement coverage

G. Overqualification of function

H. Other : ______________________________

05. If a neuro-navigation system is available, would you be interested in using it?

A. Yes

B. No

06. What do you think are the barriers to the use of navigation systems in clinical practice? (Select up to 3)

A. Device cost

B. Technical difficulty

C. Lack of reimbursement coverage

D. Lack of clinical evidence of effectiveness

E. Other : ______________________________

07. What do you think is an acceptable budget for a neuro-navigation system? (₩, WON)

A. ~10 million

B. $10 million to $50 million

C. $50 million to $100 million

D. More than 100 million

E. Other : ______________________________

08. What do you think is a more appropriate format for rTMS device and neuro-navigation system?

A. rTMS equipment and neuro-navigation are separate and can be purchased separately.

B. rTMS equipment and neuro-navigation are implemented as one system.

09. Please comment on any other unmet needs of rTMS treatment for stroke patients.

: ___________________________________________________
