# Supplementary material for: Clinical implementation, barriers, and unmet needs of rTMS and neuro-navigation systems in stroke rehabilitation: a nationwide survey in South Korea
Source: Front Neurol. 2024 Jul 30;15:1423013. doi: 10.3389/fneur.2024.1423013 (PMC11321079; doi:10.3389/fneur.2024.1423013)

Supplementary Material

# Supplementary Material 2. Survey results

**Section 1. Baseline demographics**

| **Demographics** |  | **Total (n=122)** |
| --- | --- | --- |
| Sex | Male | 88 (72.1) |
|  | Female | 34 (27.9) |
| Age | 30-39 | 50 (41.0) |
|  | 40-49 | 48 (39.3) |
|  | 50-59 | 24 (19.7) |
| Type of hospital | Designated rehabilitation hospital | 58 (47.5) |
|  | Tertiary hospital | 40 (32.8) |
|  | Rehabilitation hospital | 11 (9) |
|  | General hospital | 10 (8.2) |
|  | Clinic | 3 (2.5) |
| Job Title | Employed physicians | 71 (58.2) |
|  | Professor | 40 (32.8) |
|  | Self-employed physicians | 9 (7.4) |
|  | Fellowship | 2 (1.6) |
| Year of Specialization | 0-10 years | 67 (54.9) |
|  | 10-20 years | 37 (30.3) |
|  | Over 20 years | 18 (14.8) |

All data are presented as n(%)

**Section 2. rTMS Survey Results**

01. Have you ever applies the rTMS for patients with stroke, and if so, for how long?


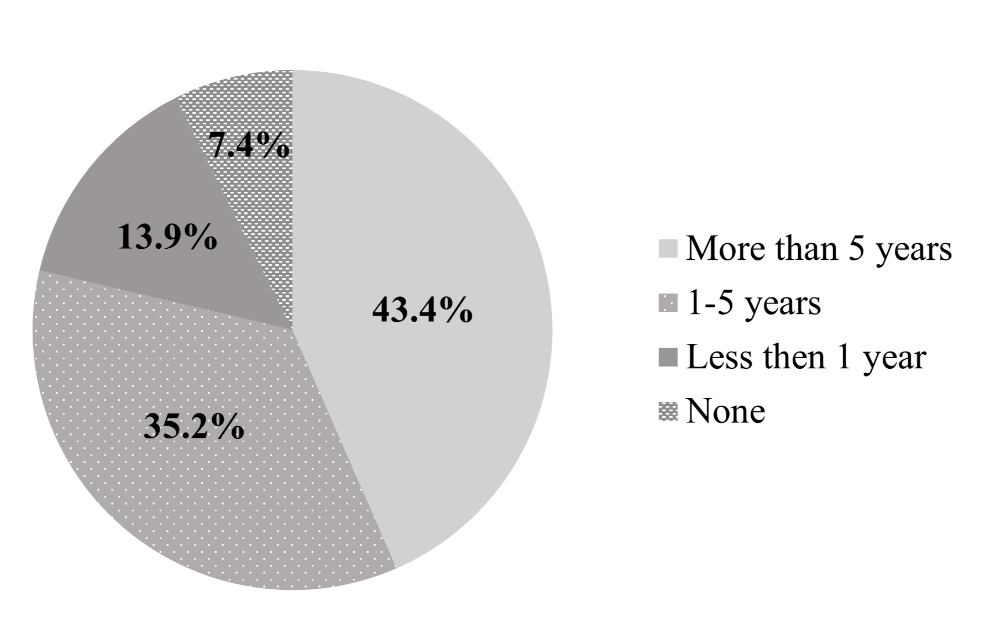


02. Do you think rTMS is effective for patients with stroke?


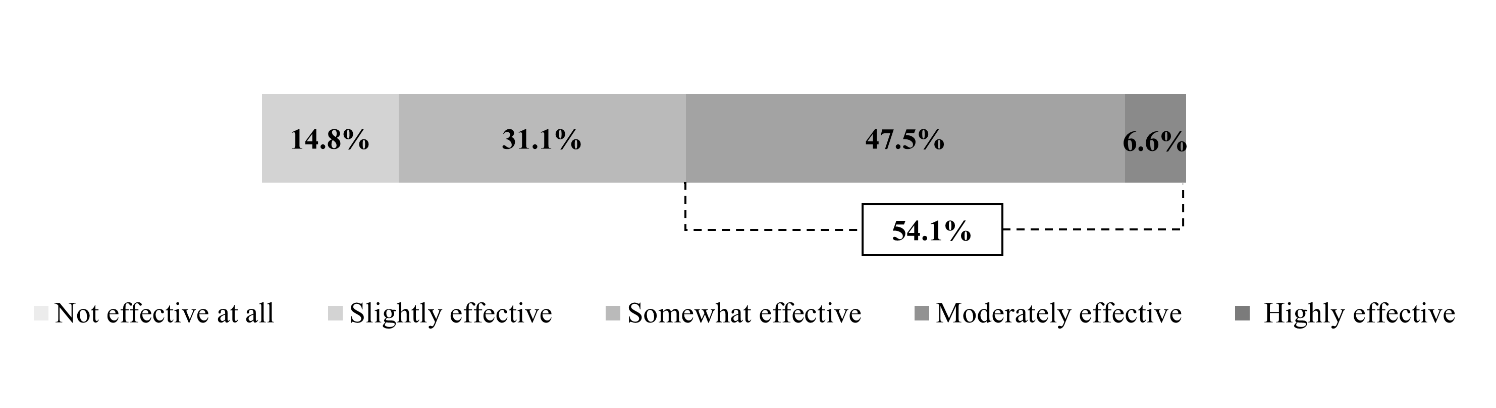


03. Are you currently applying for the rTMS for patients with stroke?


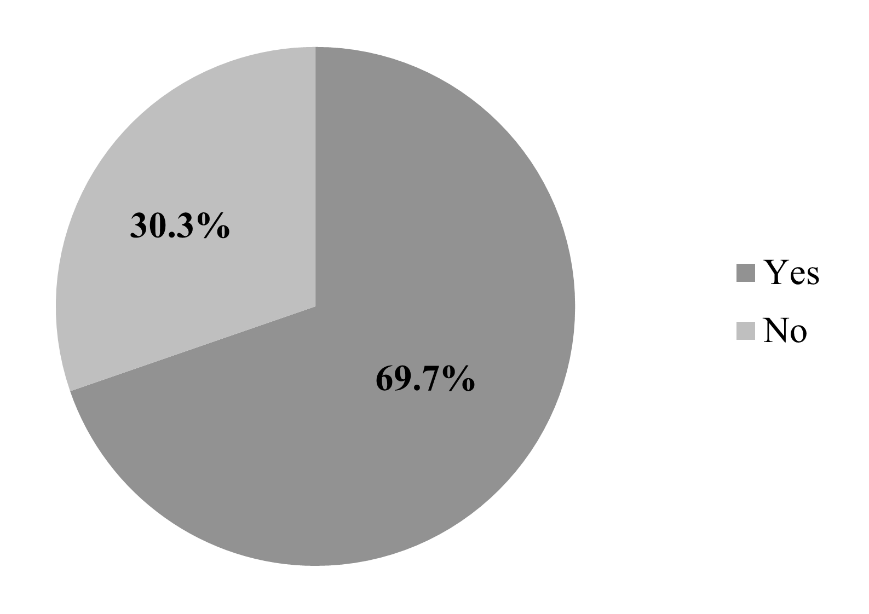


04. How do you determine the motor threshold and stimulation intensity for rTMS treatment?

*motor threshold: The minimum magnetic stimulation intensity required to produce a motor-evoked potential.


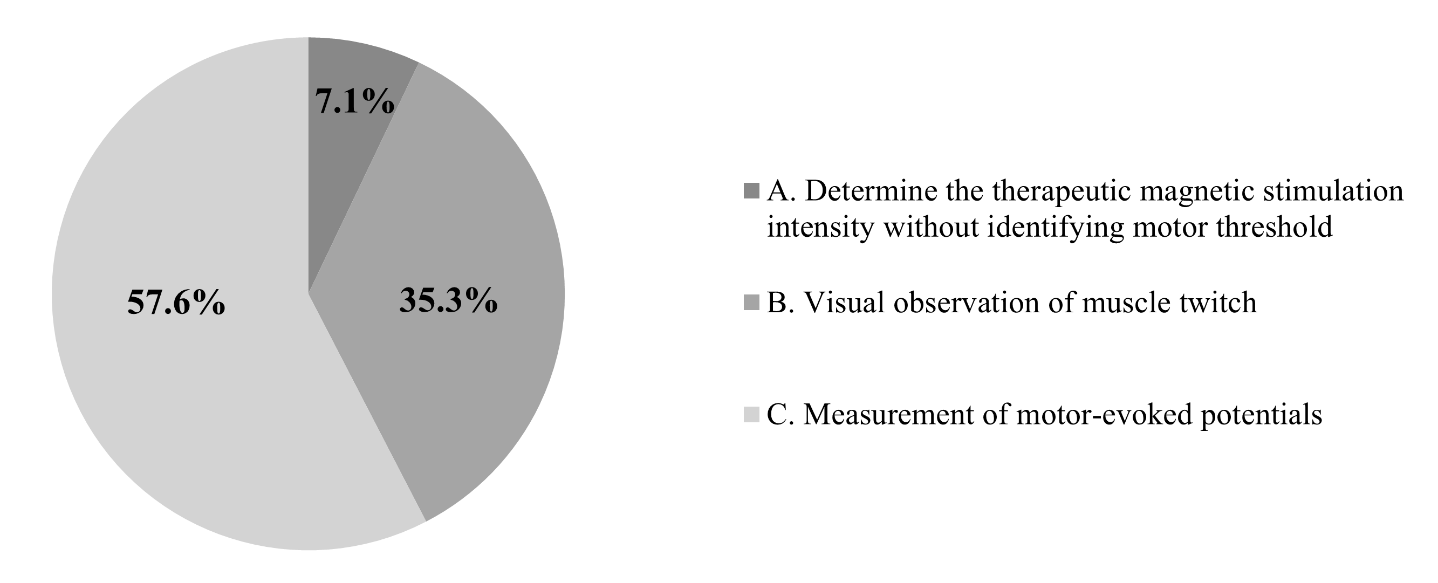


05. How do you determine the motor hot spot?

*Motor hot spot: The area of the motor cortex that, when stimulated, produces the maximum amplitude, shortest latency, and most reliable motor-evoked potential (MEP) in a specific muscle.


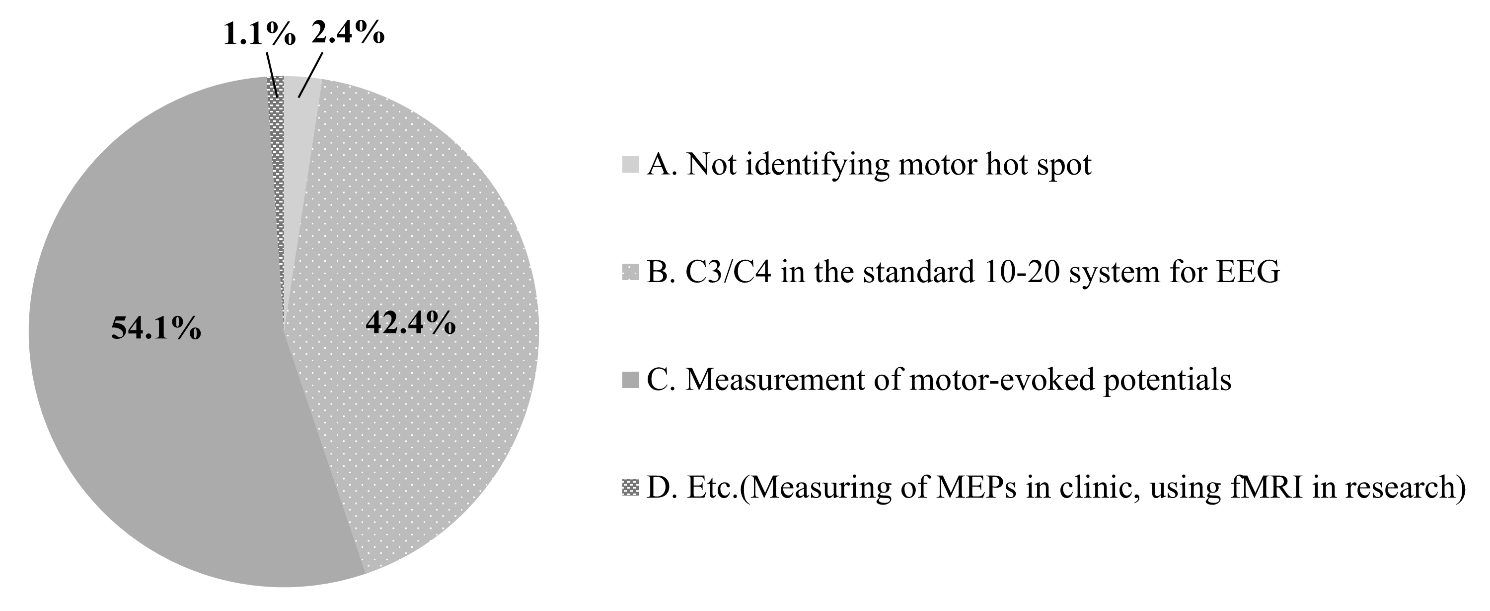


06. During an rTMS treatment, what method do you apply to keep the coil in the initial stimulation target?
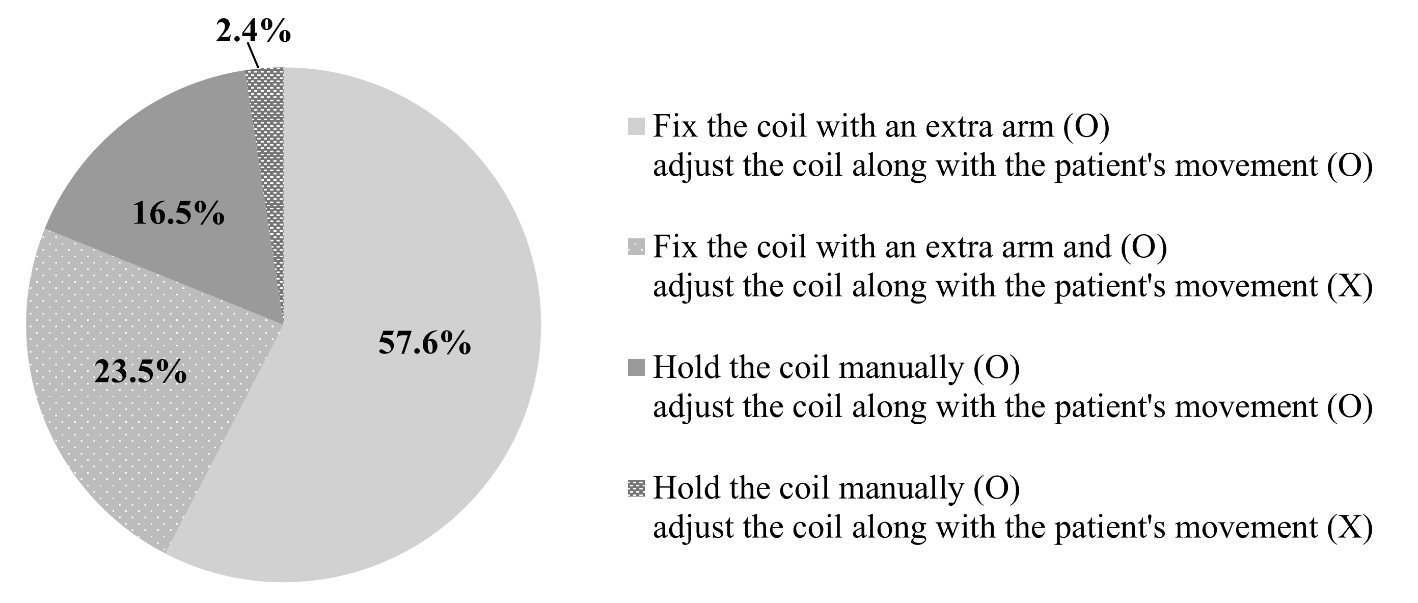


07. Do you think the magnetic coil is maintained in the initial intended location for the duration of the rTMS treatment?


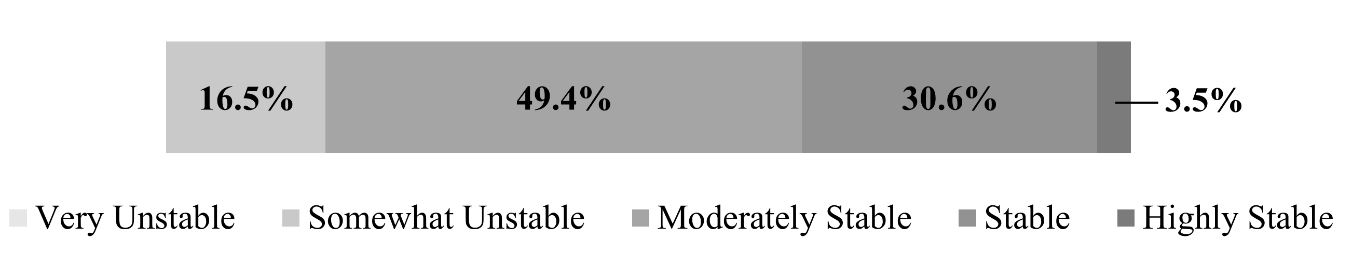


08. For which symptoms do you apply the rTMS for patients with stroke? (Select up to three)


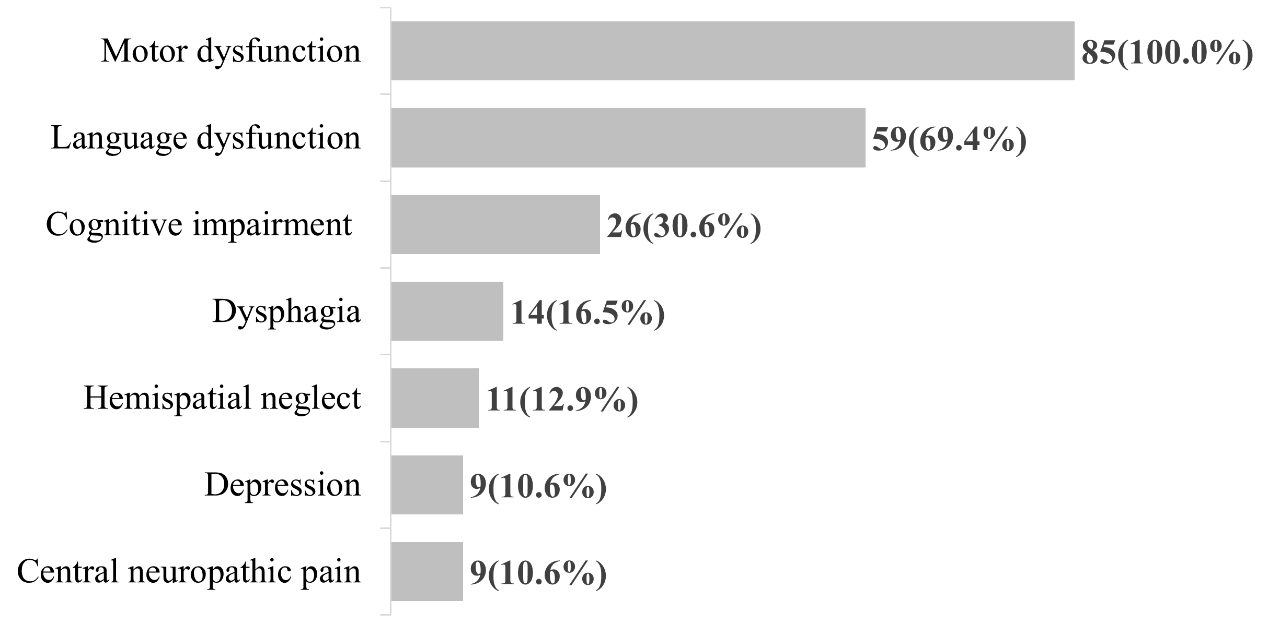


09. For which symptoms do you think rTMS is most effective for patients with stroke? (Select up to three options)


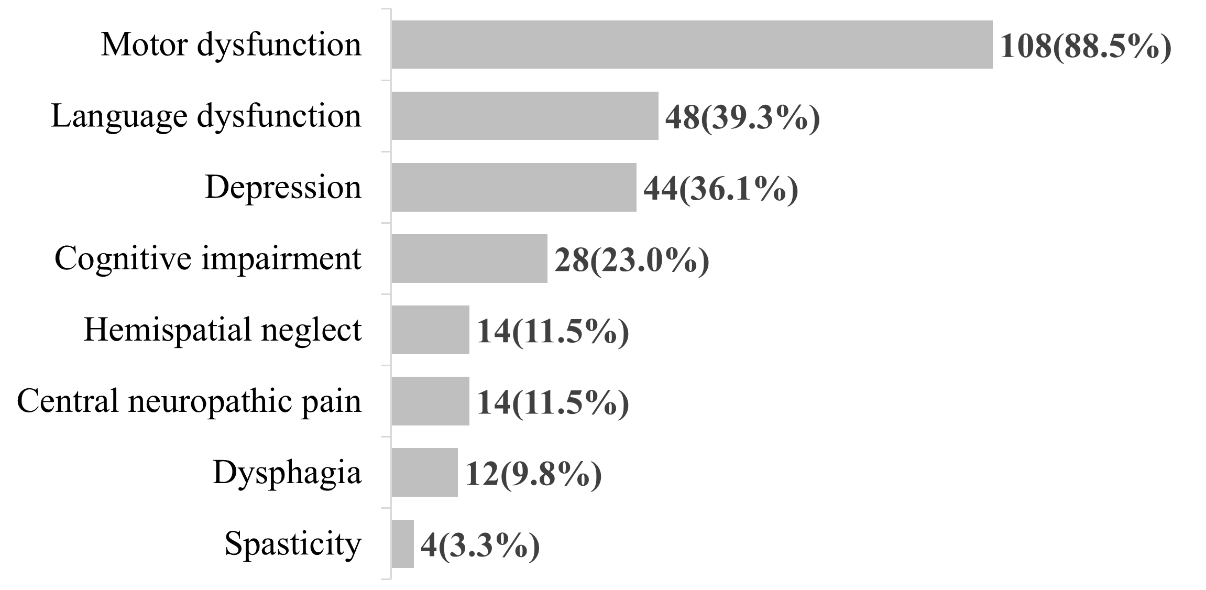


10. Does your institution have a protocol for rTMS for patients with stroke and are you familiar with it?


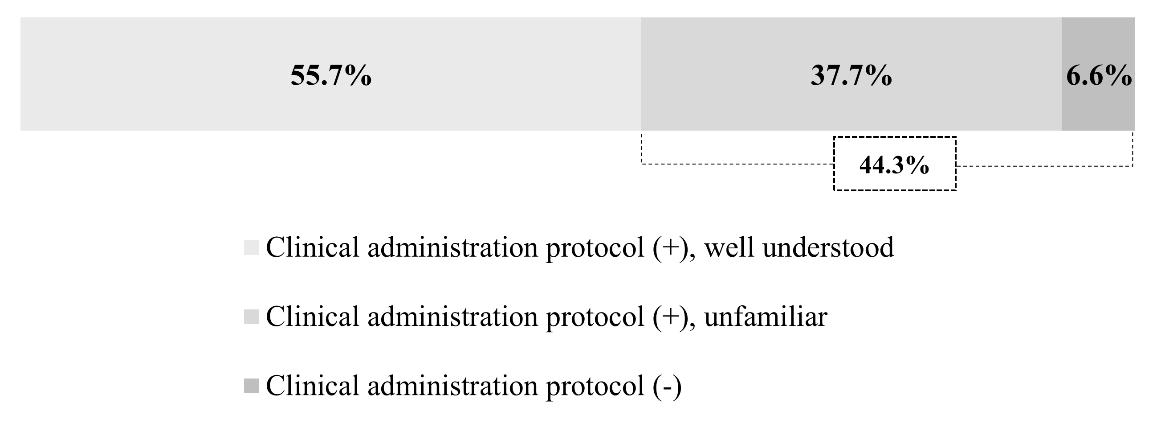


11. Which of the following parameters are difficult to determine in rTMS protocol? (Select up to three)


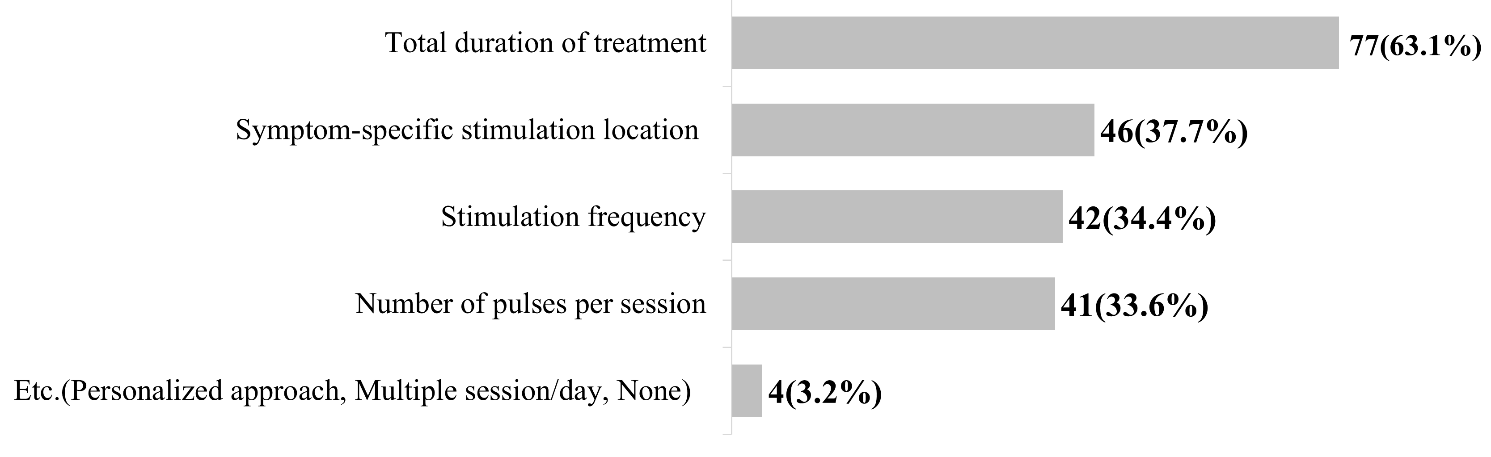


12. What are some other important considerations in rTMS protocols (e.g., disease severity, brain lesion, time since stroke onset, etc.)?


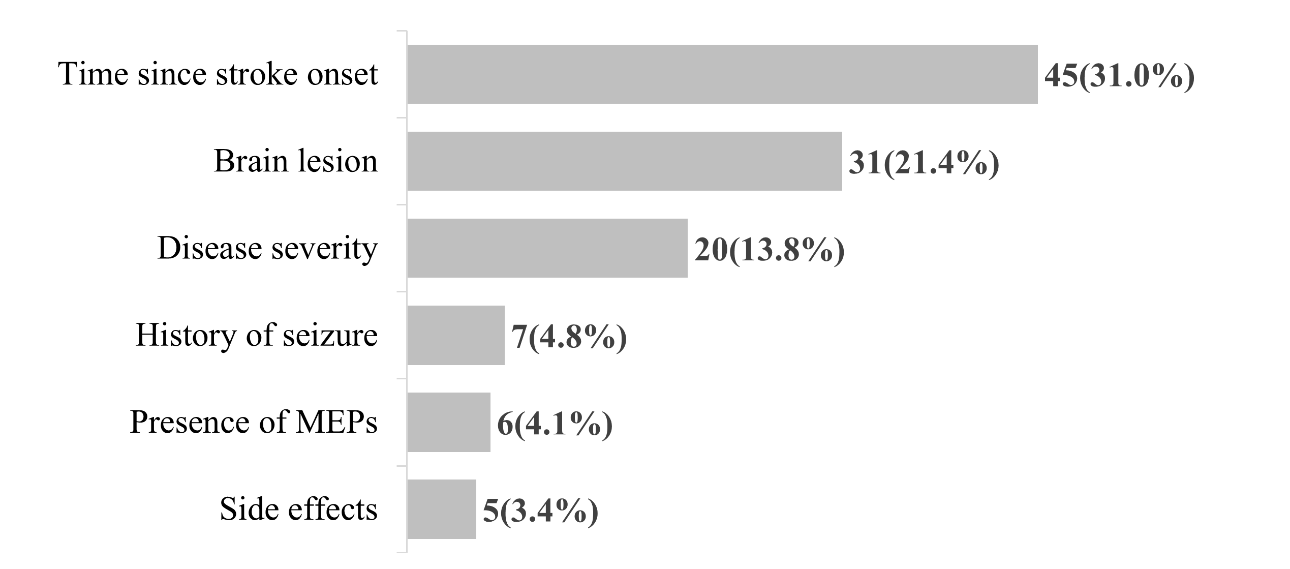


13. Do you know the major side effects of rTMS?


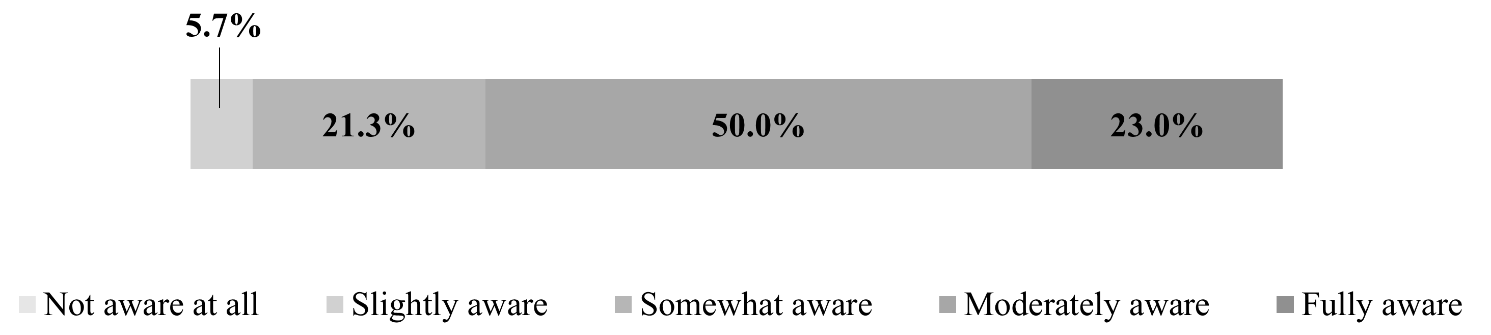


14. Which side effects are you most concerned about when applying rTMS for patients with stroke? (Select up to 3)
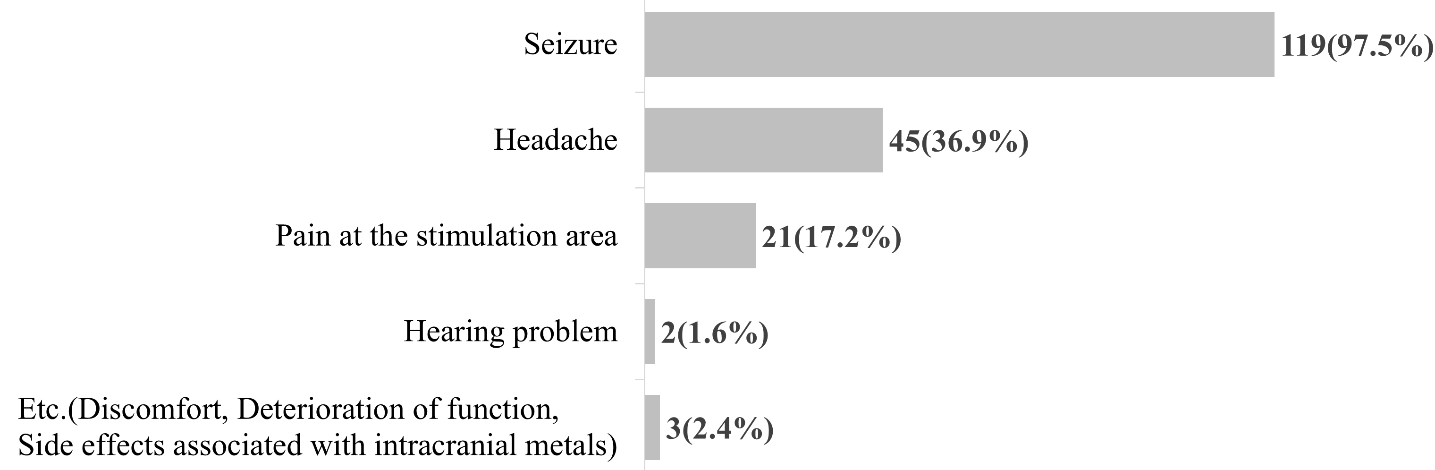


15. Do you know the contraindications of rTMS for patients with stroke?


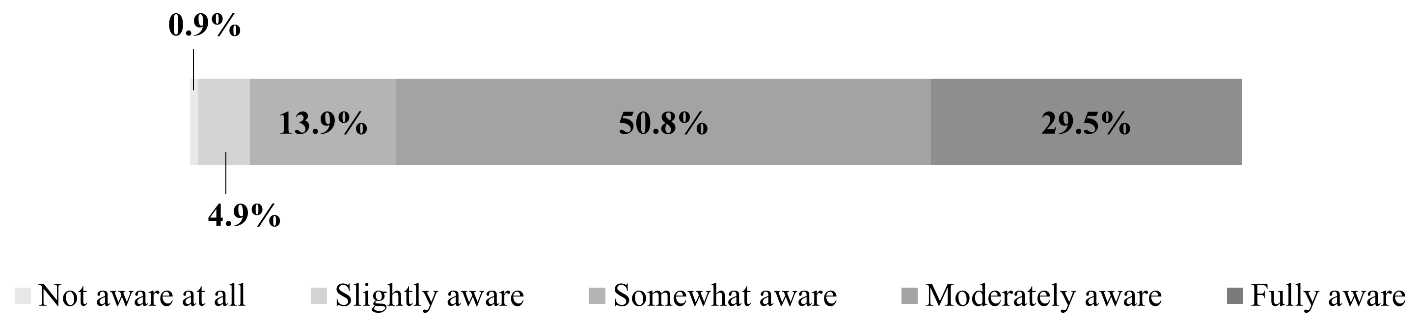


16. Do you know the safety guidelines for rTMS (e.g. the maximum number of pulses per day)?


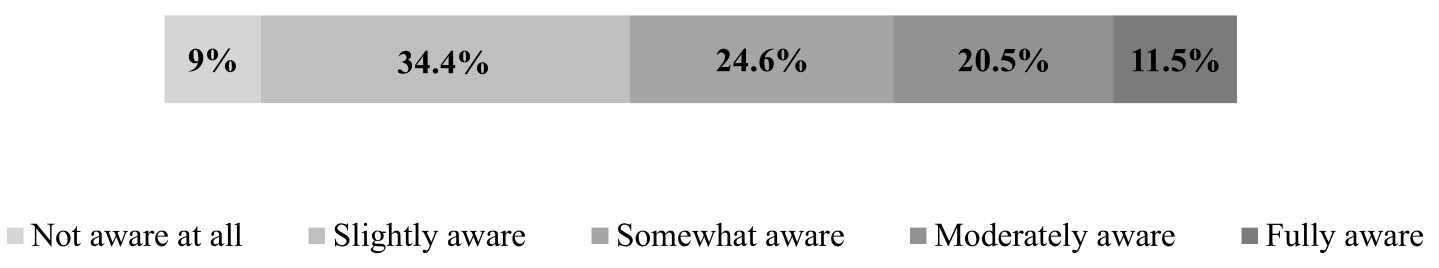


17. What are the most significant barriers to the clinical application of rTMS for patients? (Select up to 3)


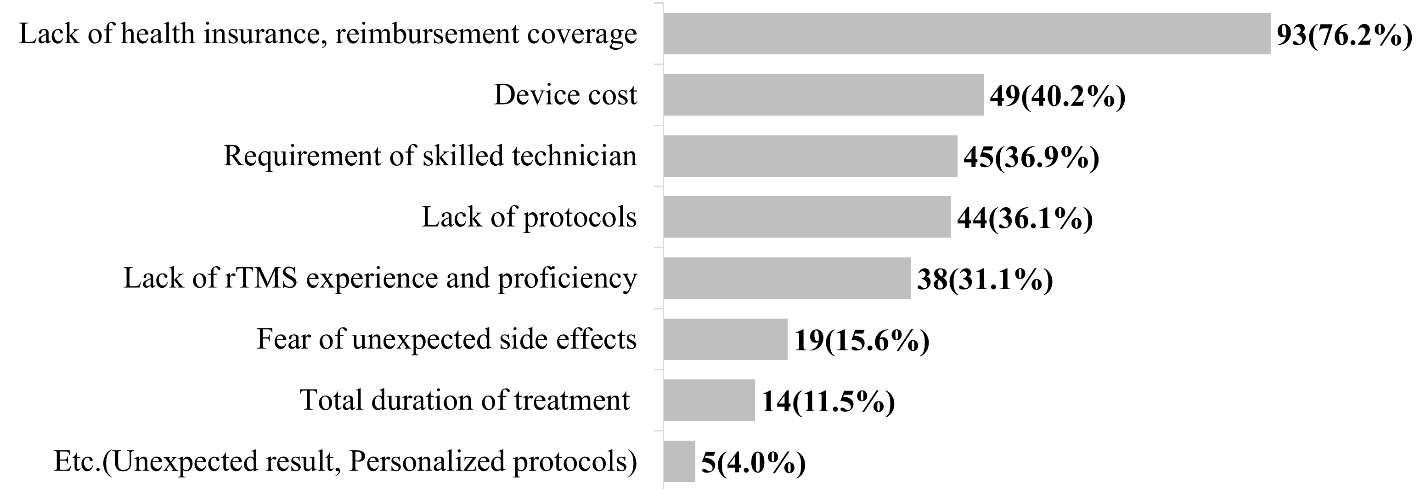


18. What is the most important consideration to increase the therapeutic effectiveness of rTMS? (Select up to 3)


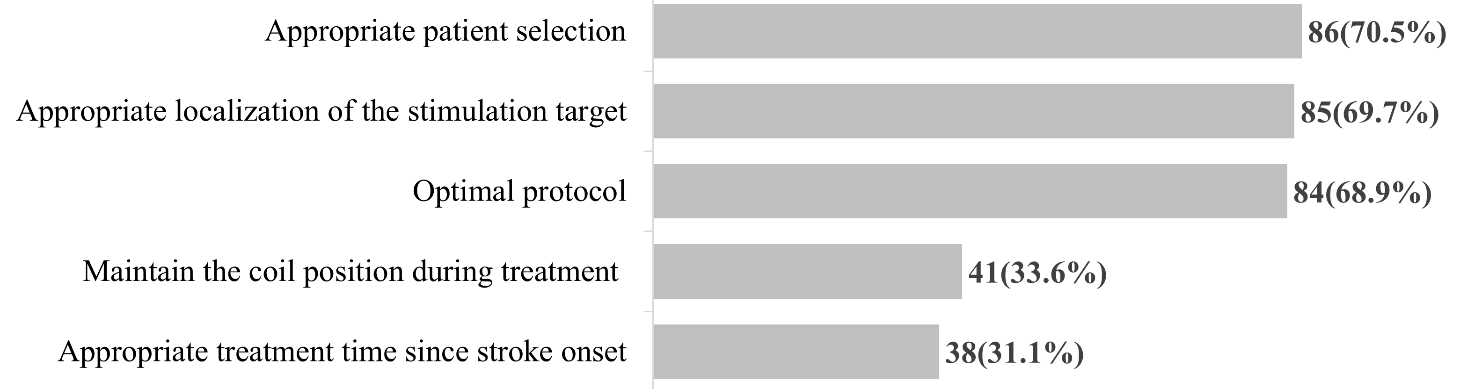


19. What needs to be improved about the rTMS device? (Select up to 3)


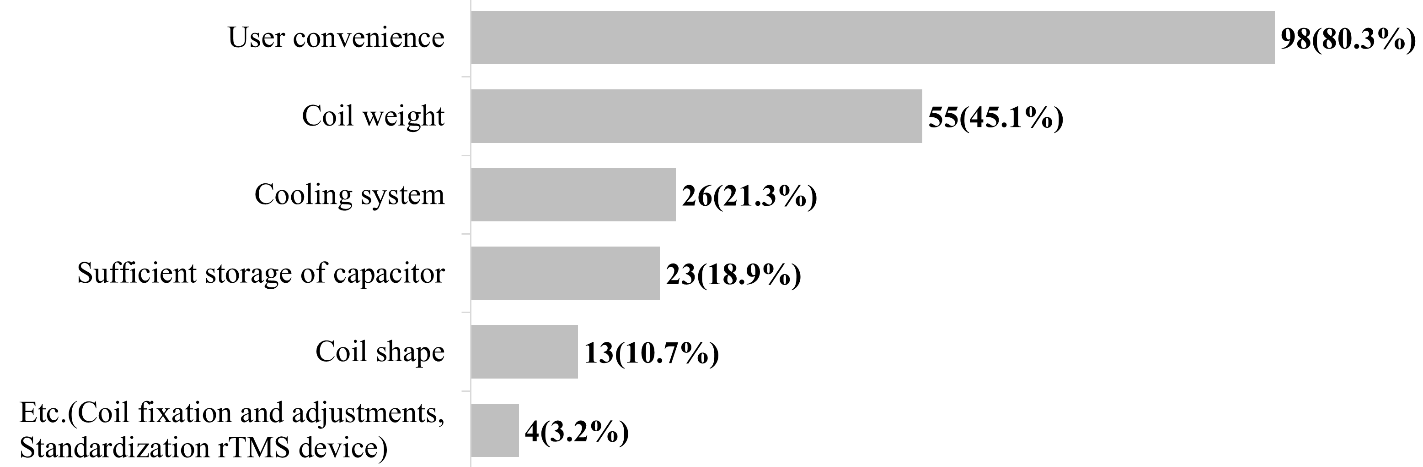


20. What do you think is an acceptable budget for an rTMS device? (₩, WON)


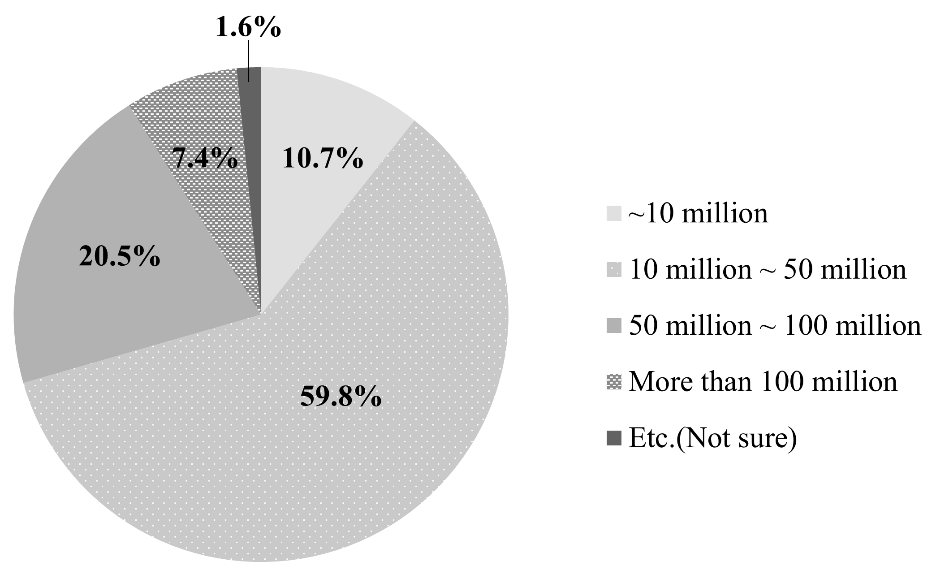


**Section 3. Survey of neuro-navigation system**

01. Are you familiar with the application of neuro-navigation in rTMS treatment?


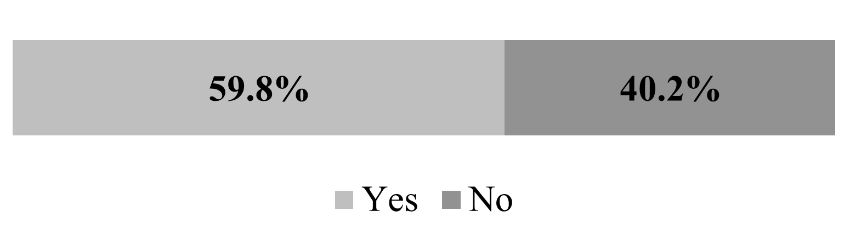


02. Do you know the concept and mechanism of the neuro-navigation system in rTMS treatment?


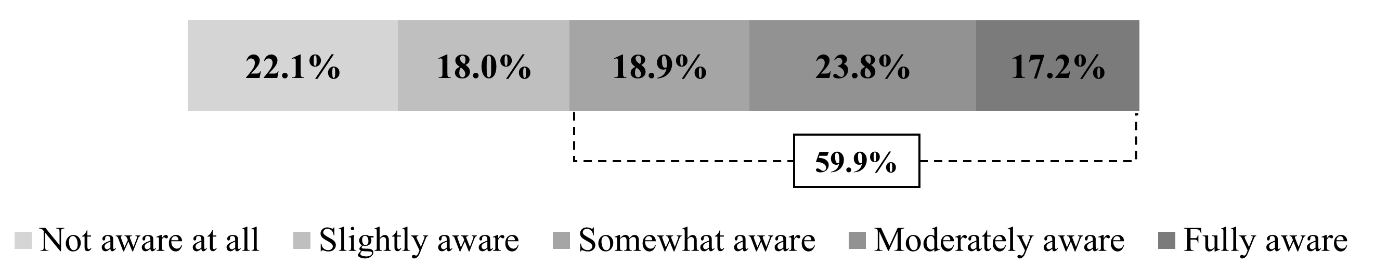


03. Does your hospital use neuro-navigation during rTMS treatment?


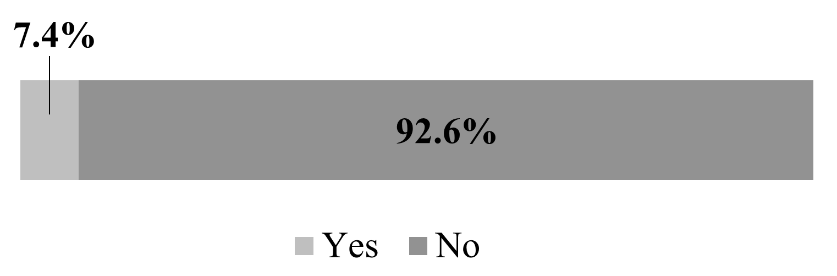


04. Which of the following do you think are the reasons for the limited use of neuro-navigation system in rTMS treatment? (select up to 4)


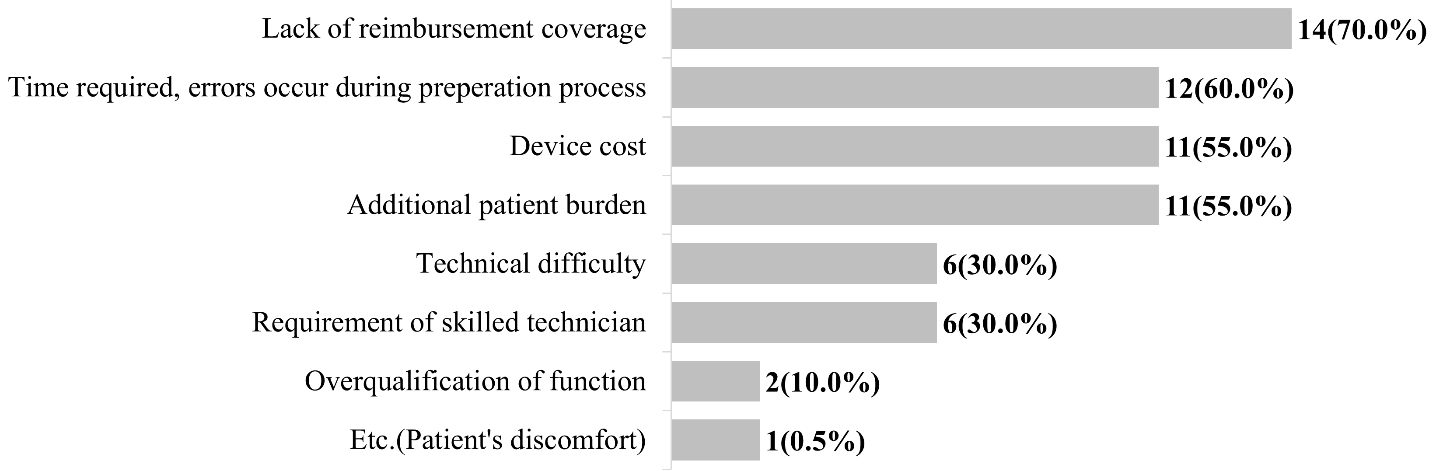


05. If a neuro-navigation system is available, would you be interested in using it?


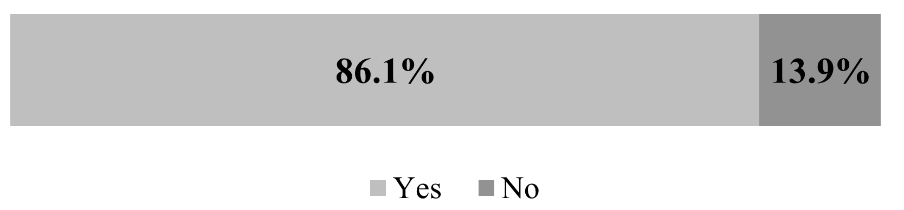


06. What do you think are the barriers to the use of navigation systems in clinical practice? (Select up to three)


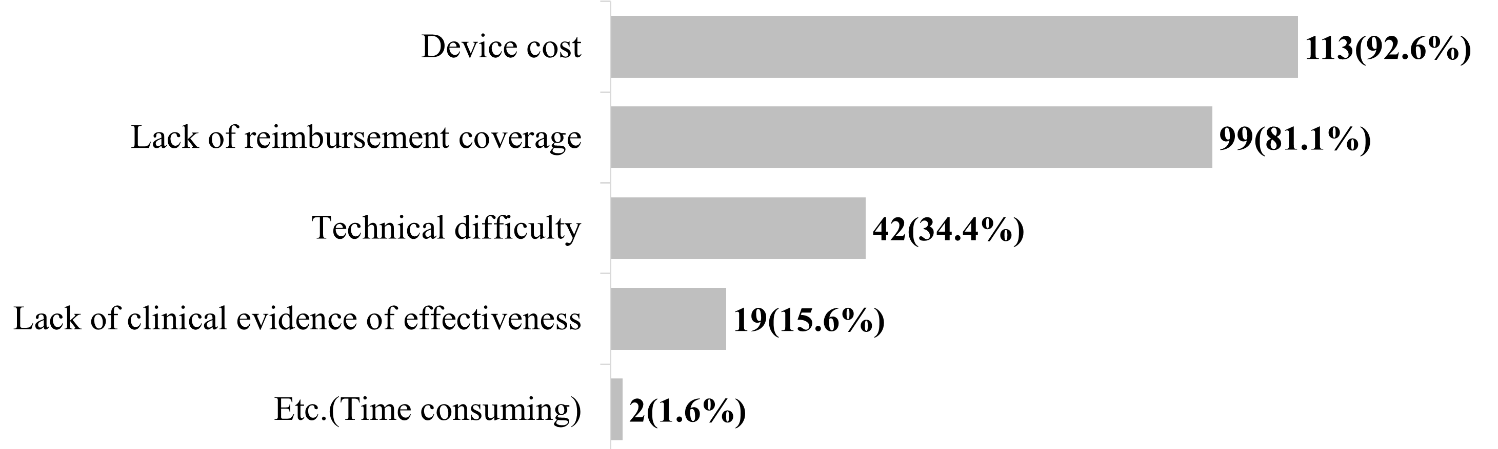


07. What do you think is an acceptable budget for a neuro-navigation system? (₩, WON)


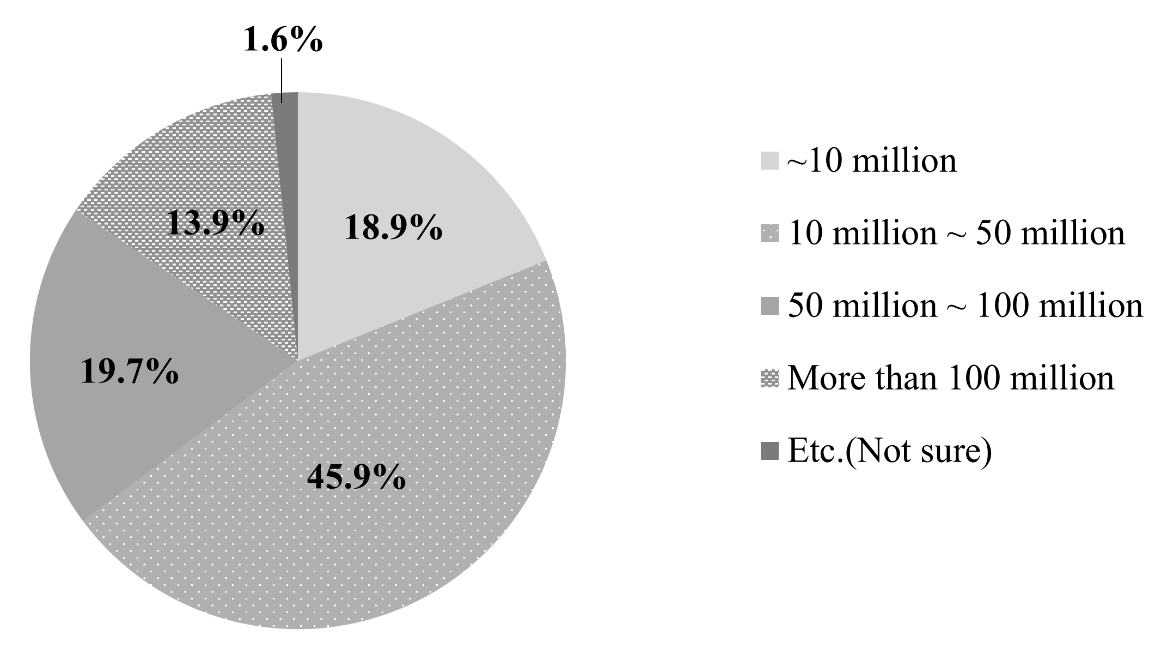


08. What do you think is a more appropriate format for a rTMS device and neuro-navigation system?


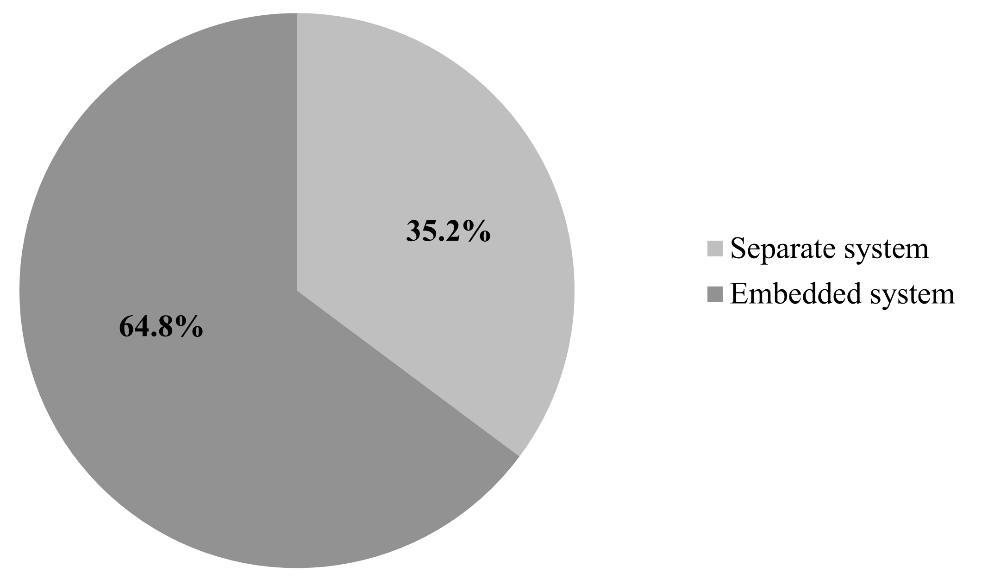


09. Please comment on any other unmet needs of rTMS treatment for patients with stroke.


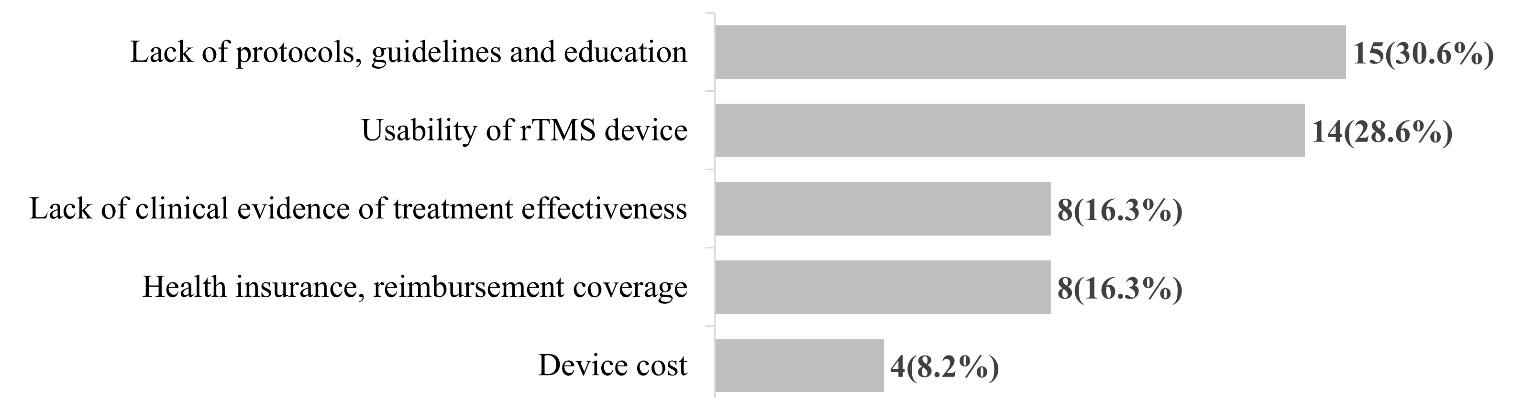

Supplement: Supplementary file 2 [file Data_Sheet_2.DOCX]
